# Fluorine-Substituent-Containing Sulfonated Poly(arylene ether) Membranes with Enhanced Proton Conductivity and Dimensional Stability for Proton Exchange Membrane Fuel Cells

**DOI:** 10.3390/molecules31173007

**Published:** 2026-08-27

**Authors:** Tung-Li Hsieh, Jia-Xian Zhang

**Affiliations:** Department of Electronic Engineering, National Kaohsiung University of Science and Technology, No. 415, Jiangong Rd., Sanmin District, Kaohsiung City 807618, Taiwan; f114152131@nkust.edu.tw

**Keywords:** sulfonated poly(arylene ether), fluorine substituent, proton exchange membrane, PEMFC, proton conductivity, microphase separation, fuel cell, dimensional stability

## Abstract

A series of fluorine-substituent-containing sulfonated poly(arylene ether) membranes was synthesized and evaluated as proton exchange membranes for fuel cell applications. Fluorinated difluoro monomers were first reacted with three different diol monomers through nucleophilic polycondensation to obtain 4FP4-series polymers, followed by controlled sulfonation to produce six S4FP4-series membranes with different ion exchange capacities and microphase-separated morphologies. FT-IR, ^1^H-NMR, and ^19^F NMR spectroscopy confirmed the chemical structures of monomers, polymers, and sulfonated polymers. The resulting polymers exhibited good film-forming ability and high thermal stability. The sulfonated membranes showed ion exchange capacities of 1.74–2.80 mmol/g, water uptake of 24.7–116.3%, and favorable dimensional stability under elevated temperature. Most S4FP4 membranes exhibited proton conductivities higher than that of Nafion 211. In particular, S4FP4a (IEC of 1.74) achieved a proton conductivity of 262 mS cm^−1^ at 80 °C and 95% RH and a maximum fuel cell power density of 1.07 W cm^−2^, outperforming Nafion 211. TEM analysis revealed that fluorine substitution promoted effective microphase separation and continuous mesoscale aggregated domain. These results demonstrate that fluorinated sulfonated poly(arylene ether)s are promising candidates for high-performance proton exchange membranes.

## 1. Introduction

**Figure 1:**
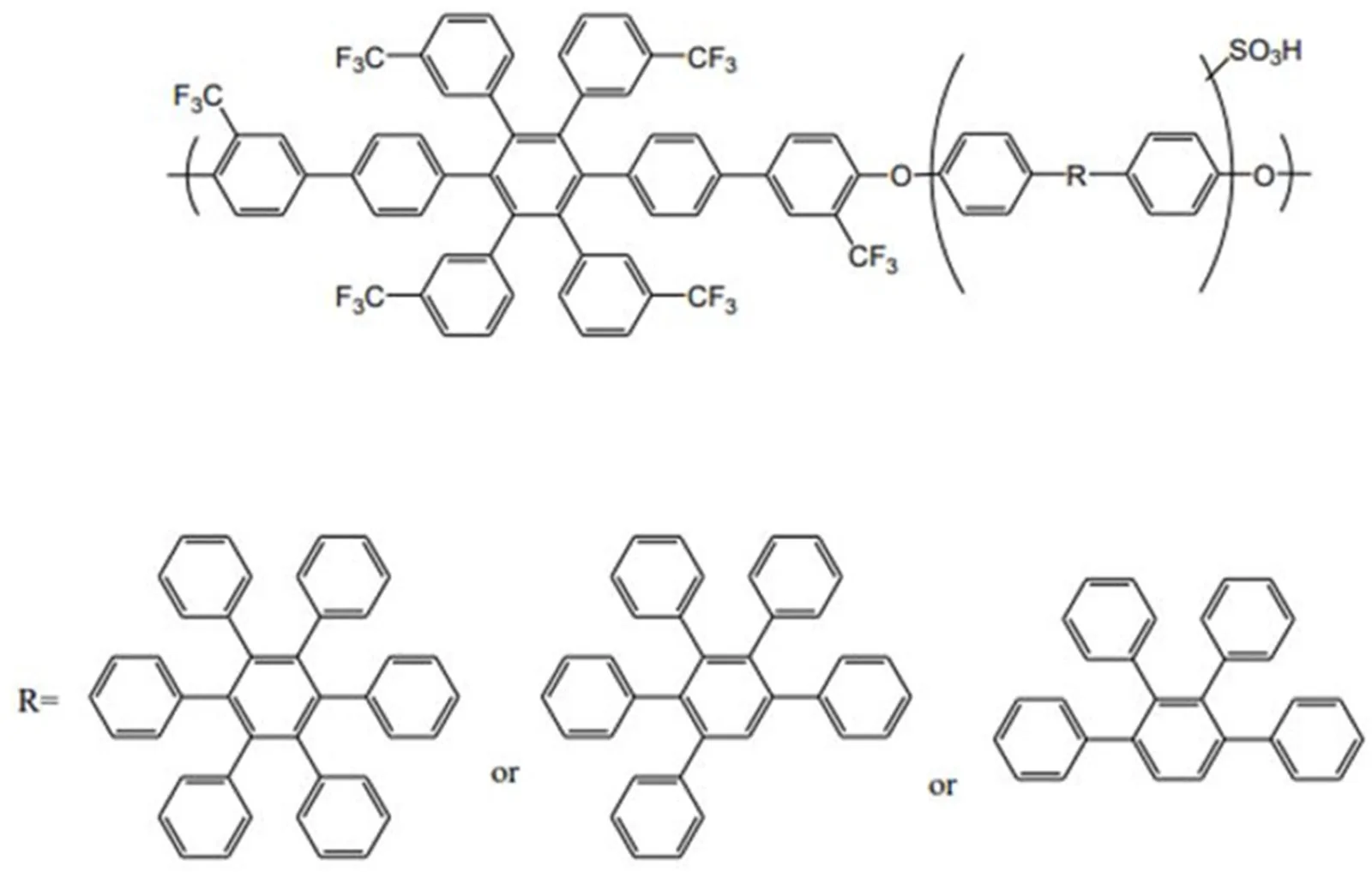
Polyfluorosulfonated polymers.

In 2014, Huang et al. developed three series of polyfluorosulfonated hydrocarbon zwitterionic polymer materials containing trifluoromethyl groups (SFA series), as shown in Figure 1. The polymer chains are composed of hydrophilic and hydrophobic segments: the hydrophobic segment is a difluoro monomer containing six trifluoromethyl groups, while the hydrophilic segment is a multi-benzene ring diol monomer. Introducing fluorine atoms can effectively enhance various properties and stability of the polymer materials, with several advantages: it (1) improves polymer solubility to increase molecular weight, enhancing the thermal stability of the polymer; (2) effectively controls the grafting positions of sulfonate groups, developing polymer membranes with localized and dense sulfonation; and (3) disrupts structural regularity due to the reduced spacing between carbon-fluorine bonds, which increases the free volume and allows easier entry for water molecules, enhancing the water uptake and water retention capacity of the polymer membranes at different temperatures. In conclusion, the introduction of fluorine atoms can effectively improve various membrane properties [1].

**Figure 2:**
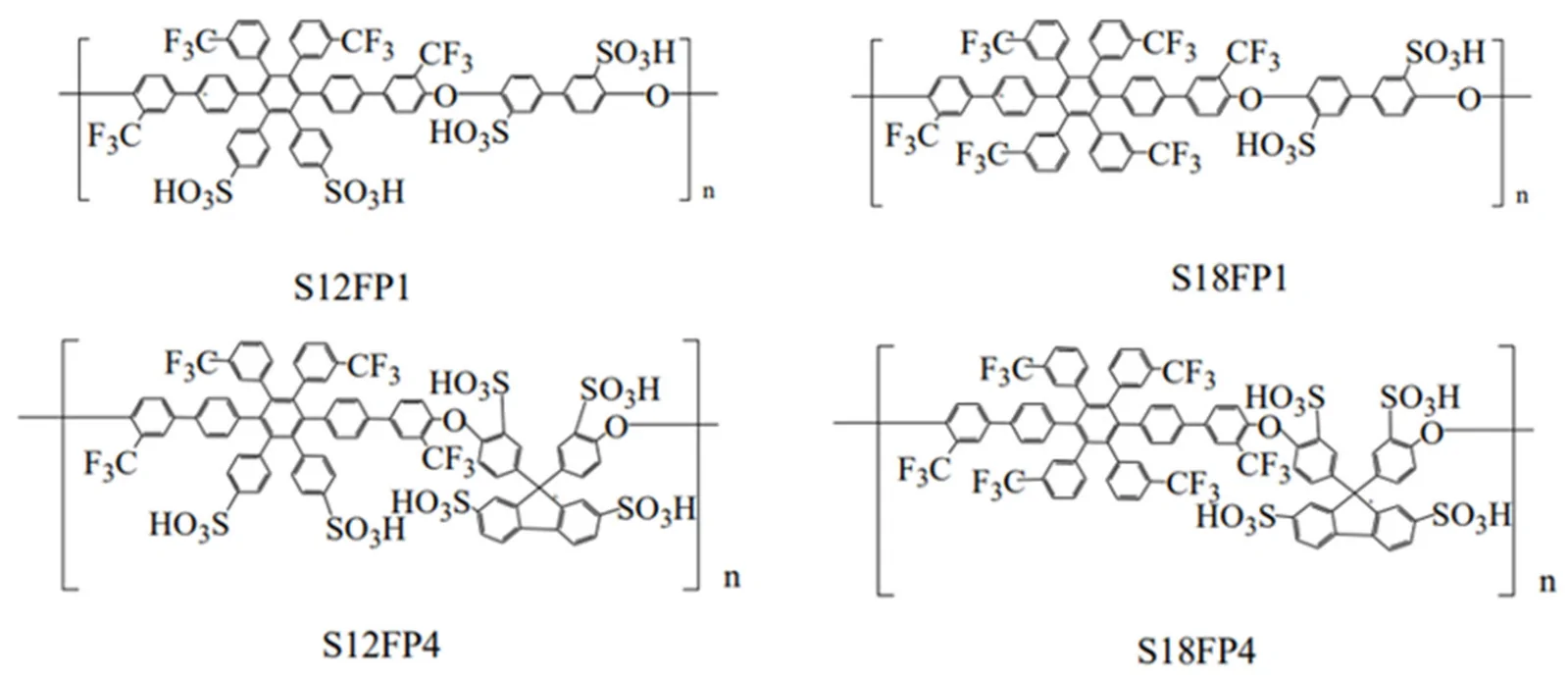
FP series polymers.

**Figure 3:**
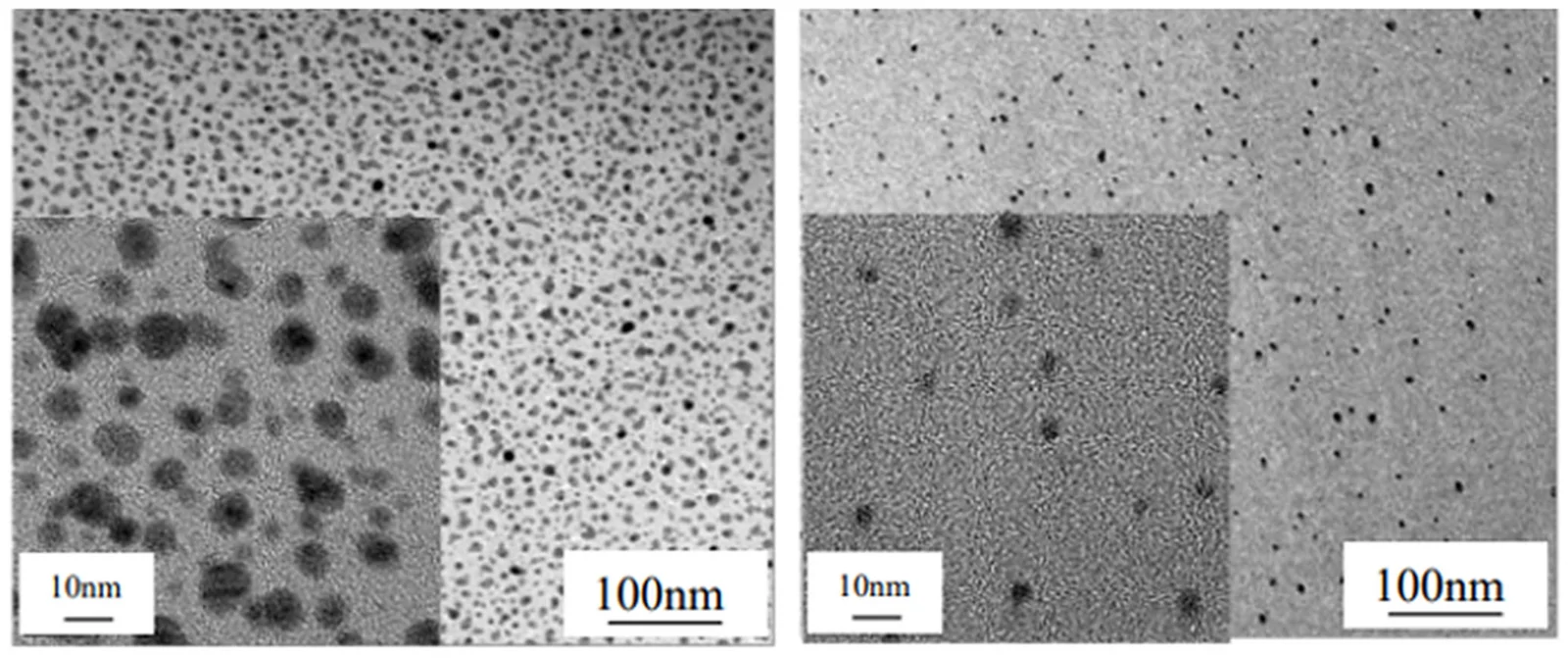
TEM micrographs of FP series polymers S12FP1 and S18FP1.

In 2015, Huang et al. developed four structural variations with alternating poly(arylene ether)s containing trifluoromethyl groups (FP series). These polymers were prepared through the polycondensation reaction of multi-benzene ring difluoro monomers with different fluorine counts and two commercially available diol monomers, as shown in Figure 2. The primary objective was to investigate whether controlling the number of CF_3_ functional groups affected the microphase separation morphology of the membranes. According to the transmission electron microscopy (TEM) images, the ion cluster size of S18FP1 ranged between 8 and 18 nm. In comparison, that of S12FP4 was approximately 10 to 23 nm, as shown in Figure 3. These observations demonstrated that a higher number of fluorine atoms led to smaller ion clusters. In contrast, fewer fluorine atoms resulted in larger ion clusters. Consequently, regulating the fluorine content can alter the quantity and position of sulfonate groups, affecting the microphase separation morphology of the membranes.

**Figure 4:**
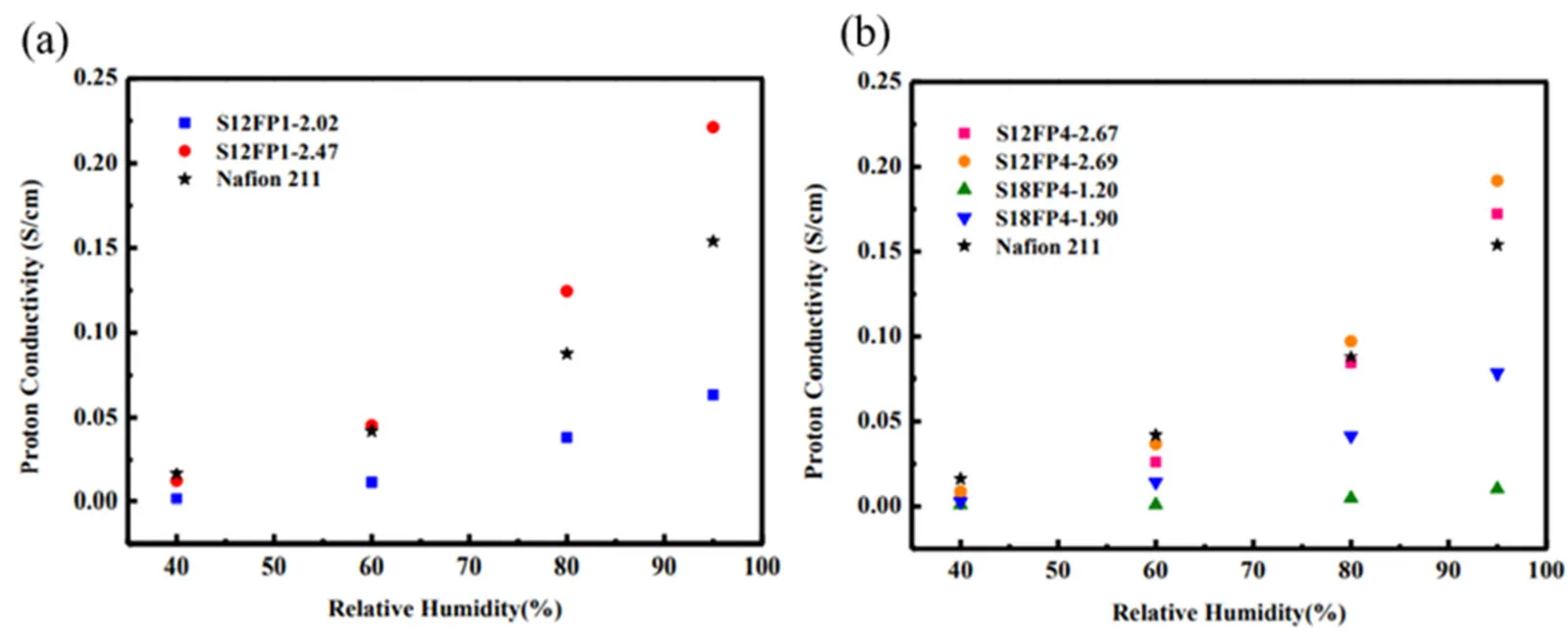
Proton conductivity of (**a**) S12FP1, and Nafion 211; (**b**) S12FP4, S18FP4, and Nafion 211.

**Figure 5:**
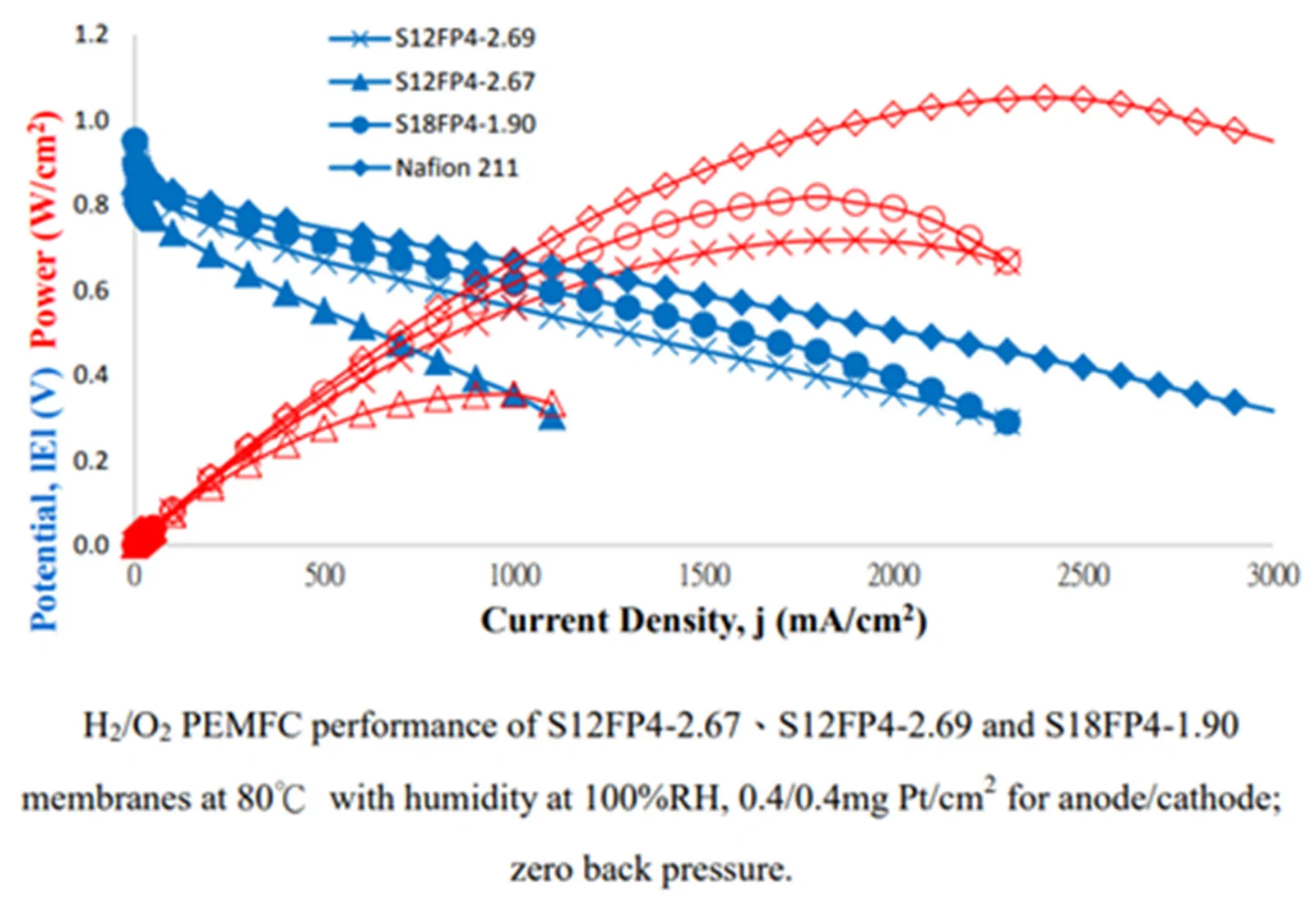
Device efficiency of S12FP4, S18FP4, and Nafion 211.

Experimental data indicate that increasing the number of fluorine atoms can effectively restrict the grafting positions and quantities of sulfonate groups, affecting the water uptake of the membranes. At a high temperature (90 °C), the water uptake among S12FP1, S12FP4, and S18FP4 varied significantly (13–162%), although their dimensional change rates showed minimal difference (6–35%). Therefore, it is speculated that the introduction of fluorine allows the sulfonated membranes to retain high proton conductivity due to high water uptake while simultaneously maintaining good dimensional stability, as shown in Figure 4. Furthermore, introducing fluorine atoms can also increase material solubility to enhance reactivity, increasing the molecular weight of the polymer. However, the results demonstrated that high proton conductivity is not necessarily proportional to device efficiency; the polymer with the optimal device efficiency in this series was S18FP4-1.90 (0.82 W/cm^2^), as shown in Figure 5 [2].

**Figure 6:**
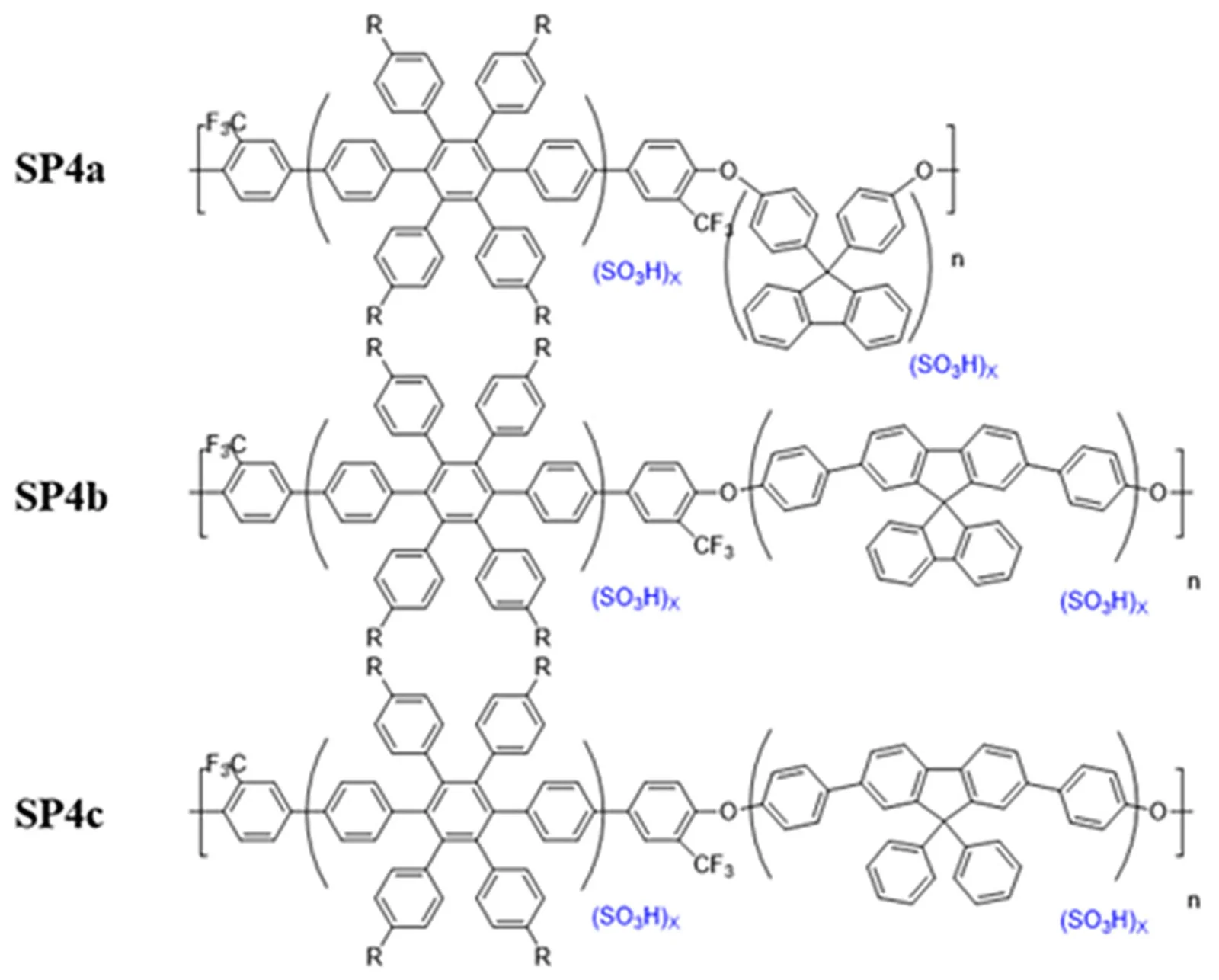
Schematic diagram of polymer design.

This study is based on multi-phenyl ring poly(arylene ether) polymers (SP4 series), as shown in Figure 6. This material was synthesized through the polycondensation reaction of a multi-phenyl ring difluoro monomer possessing high steric hindrance with three types of diol monomers. Experimental data indicate that the SP4 series exhibits excellent water uptake and proton conductivity; notably, the proton conductivity of SP4a-2.73 can reach as high as 264 mS/cm at 80 °C and 95% RH. However, due to the high water uptake of this polymer series, its dimensional stability is extremely poor. At moderate-to-high degrees of sulfonation, severe membrane swelling leads to water dissolution at room temperature and 95% RH, which poses significant challenges for its application in fuel cell devices.

According to previous work, introducing fluorine atom groups into the polymer backbone can substantially improve the properties of polymer membranes. Due to the highly hydrophobic nature of fluorine atoms, the dimensional stability of the membranes can be effectively enhanced. Additionally, introducing CF_3_ into a polymer exerts effective control over the grafting of sulfonate groups, and an increase in CF_3_ content may shrink the ion cluster channels formed by these sulfonate groups, altering the microphase separation morphology of the membranes. Although experimental data confirm that introducing CF_3_ effectively improves dimensional stability, its hydrophobic nature results in lower water uptake. Furthermore, because CF_3_ acts as a strong electron-withdrawing group, it hinders the grafting of sulfonate groups and consequently reduces the degree of sulfonation. This leads to a decrease in proton conductivity, sometimes to levels too low to be measured. Therefore, while introducing excessive fluorine groups can substantially enhance dimensional stability, it also introduces drawbacks such as an excessively low ion exchange capacity (IEC) value, insufficient water uptake, and poor proton conductivity.

The S4FP4 series prepared in this study serves as an extension of the SP4 series, wherein fluorine substituents are introduced onto the difluoro monomer within the backbone structure to investigate the effects of fluorine substitution on the characteristics of multi-phenyl ring poly(arylene ether) membranes. Compared to the trifluoromethyl group, a single fluorine atom does not possess a highly hydrophobic nature. Therefore, it is anticipated that the introduction of mono-fluorine substituents can effectively enhance dimensional stability while retaining the original water uptake of the polymer. Moreover, because the hydrophobic difluoro monomers of the backbone in the S4FP4 series are fully substituted with fluorine atoms, the sulfonate groups can be densely distributed on the hydrophilic diol monomers. This configuration establishes distinct hydrophilic/hydrophobic segments, leading to a well-defined microphase separation morphology. Therefore, this approach is expected to effectively boost both the proton conductivity and device efficiency of the polymer. Additionally, the higher cost of trifluoromethyl groups compared to mono-fluorine is a disadvantage for commercialization. This study aims to fabricate fuel cell components that perform comparably to or better than the SP4 series at a lower cost [3,4,5].

## 2. Materials and Methods

### 2.1. Preparation of Polymer Membranes

**Figure 7:**
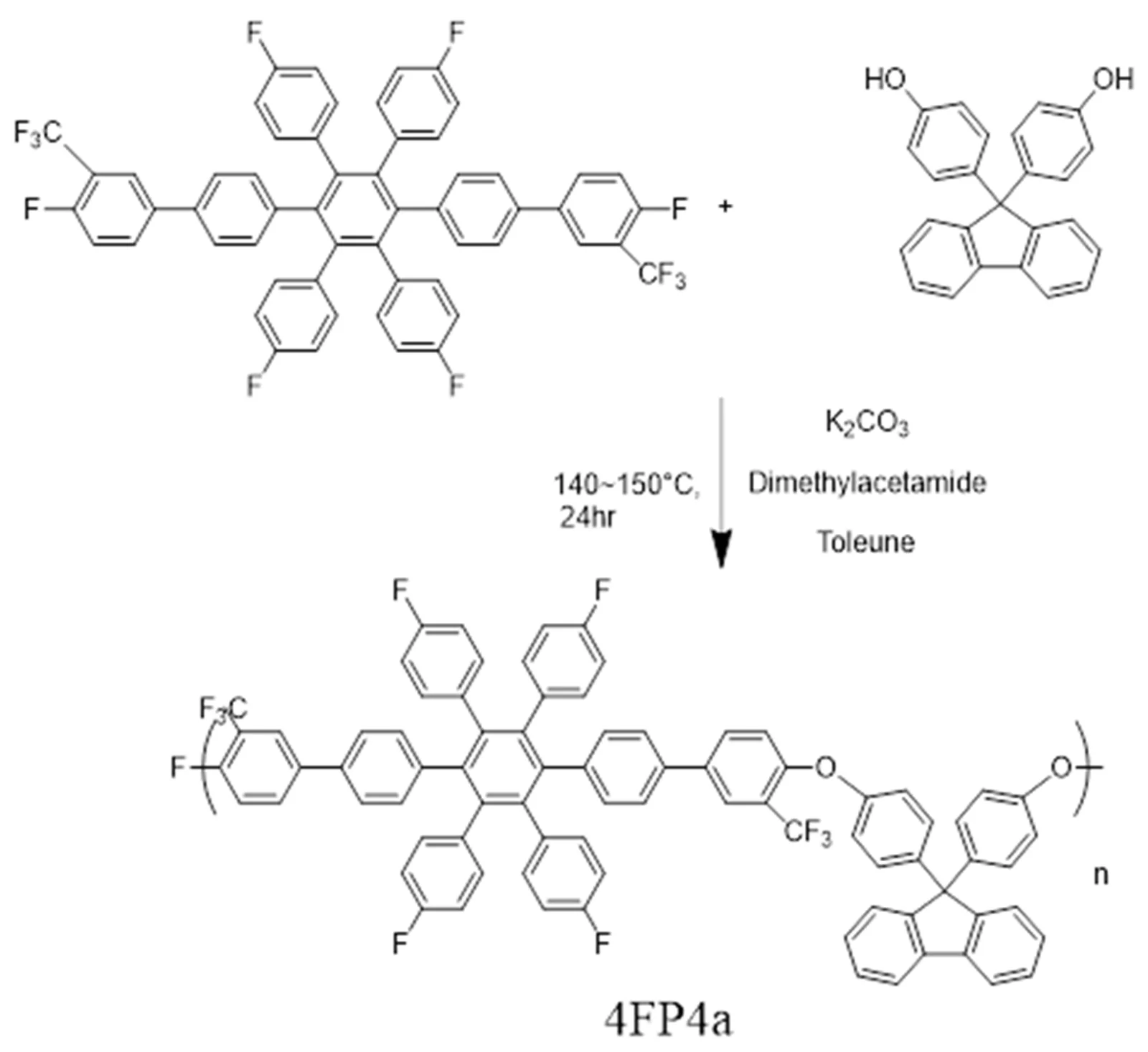
Chemical structure of 4FP4a polymer.

**Figure 8:**
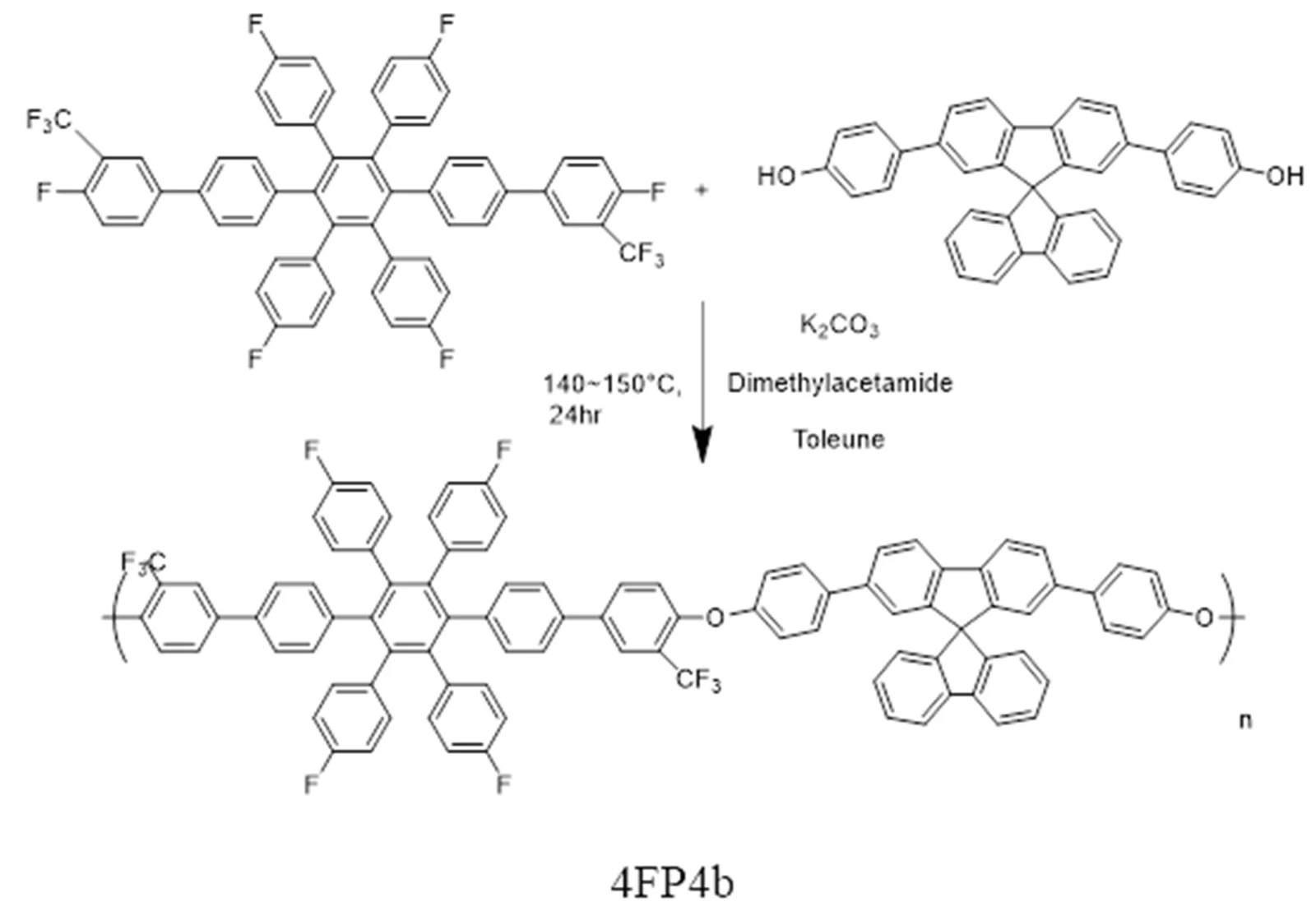
Chemical structure of 4FP4b polymer.

**Figure 9:**
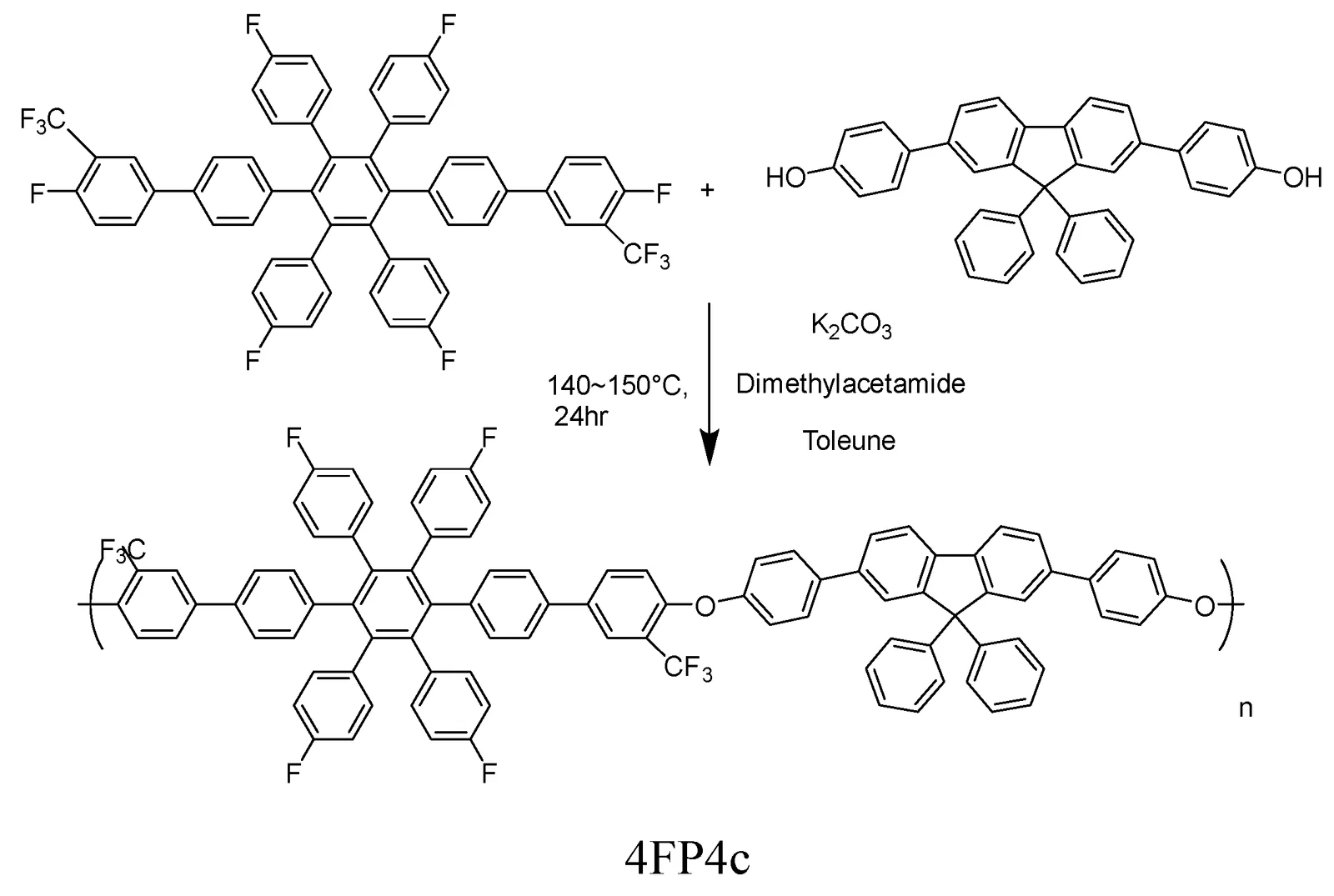
Chemical structure of 4FP4c polymer.

First, the polymers 4FP4a (Figure 7), 4FP4b (Figure 8), and 4FP4c (Figure 9) were dissolved separately in dimethylacetamide (DMAc) to achieve a concentration of 7 wt%. The mixtures were then placed in an oven and heated at 50 °C for 24 h and removed once complete dissolution was achieved. Subsequently, impurities in the materials were removed via suction filtration. The resulting filtrate was cast onto a glass substrate and spread evenly using a coating rod. It was then placed in an oven and baked at 70 °C for 8 to 12 h to form membranes. After cutting the membranes into appropriate sizes, they were pressed flat with glass plates and finally dried in a vacuum oven at 70 °C [6,7].

### 2.2. Polymer Sulfonation

A 100-mL two-necked reaction flask was charged with lauric acid (1.2 eq.) and 30 mL of anhydrous dichloromethane. A nitrogen needle was then inserted to purge the flask with nitrogen gas. Under a continuous nitrogen atmosphere, the lauric acid solution was stirred uniformly using a magnetic stir bar until complete dissolution was achieved. After the lauric acid was completely dissolved, chlorosulfonic acid (1 eq.) was introduced into a pressure-equalizing dropping funnel and then slowly added dropwise to the lauric acid solution while stirring continuously for approximately 1 h. Subsequently, 1.2 g of the polymer (previously dried in a vacuum oven for 6 h) and 200 mL of anhydrous dichloromethane (completely dehydrated by distillation before use) were added to a 500-mL three-necked round-bottom flask. A magnetic stir bar was added, and the mixture was stirred uniformly under a nitrogen atmosphere for approximately 12 h. Once the polymer was completely dissolved, the prepared sulfonating reagent was transferred into the pressure-equalizing dropping funnel via a double-tipped needle. The dropping funnel was then used to slowly add the sulfonating reagent dropwise into the fully dissolved polymer solution. Finally, a nitrogen balloon was attached to ensure that the internal environment remained saturated with nitrogen gas throughout the reaction, which proceeded for approximately 24 h.

Upon completion of the reaction, the reaction mixture was poured into a 1:1 (*v*/*v*) mixture of ethyl ether and toluene to induce precipitation and solidification while washing away the lauric acid. The solidified sulfonated polymer was collected via suction filtration. The obtained sulfonated polymer was then immersed in deionized (DI) water for washing and subsequently dried in an oven. Next, the crude product was re-introduced into a 1:2 (*v*/*v*) mixture of toluene and ethyl ether and stirred. After another suction filtration, the material was washed again with DI water. The aforementioned precipitation and washing steps were repeated several times until the lauric acid was thoroughly removed from the product and the washing solvent reached neutrality. Finally, the sulfonated polymer was isolated by suction filtration and dried in a vacuum oven at 80 °C for 12 h. Table 1 presents the sulfonating reagents at different concentrations used to sulfonate the polymers according to these ratios, yielding two types of polymers with different degrees of sulfonation [8,9,10].

### 2.3. Membrane Preparation and Acid Exchange

**Figure 10:**
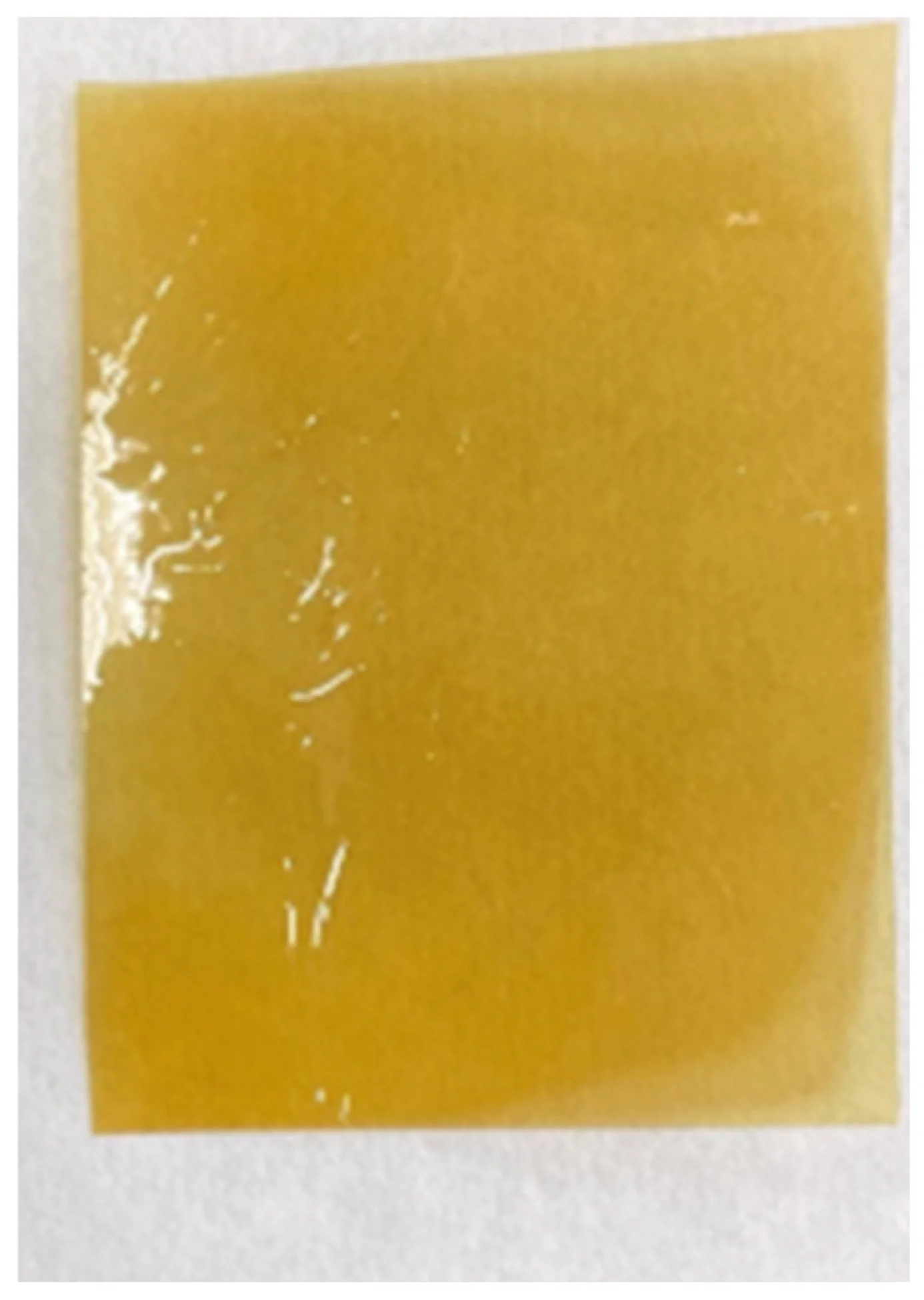
S4FP4 series membrane.

First, the sulfonated polymer was dissolved in dimethyl sulfoxide (DMSO) to prepare a 10 wt% casting solution. The solution was transferred into a vial equipped with a small magnetic stir bar. The vial containing the casting solution was then placed in a sand bath on a hot plate and stirred at 80 °C. Upon complete dissolution, any impurities were removed via suction filtration. After confirming that the solution was homogeneous and bubble-free, the filtered sulfonated polymer solution was cast onto a glass substrate using an automatic film coater. The cast solution was then placed in an oven at 80 °C and baked for at least 20 h to form a membrane. Subsequently, the membrane was peeled from the glass substrate and immersed in a 1 M HCl aqueous solution for more than 12 h. This acid treatment process was repeated twice to ensure complete proton exchange. Subsequently, the acid-treated membrane was removed and placed into a beaker containing deionized (DI) water, where it was washed repeatedly until the pH of the washing water reached 7.0 (neutral). The washed membrane was then placed between two sheets of filter paper to blot the excess moisture. It was then transferred between two fresh sheets of filter paper. Finally, the filter papers enclosing the membrane were sandwiched between two glass plates and placed into a vacuum oven, where the membrane was dried and formed at 80 °C under vacuum. Figure 10 shows the final sulfonated polymer membrane [11,12,13].

### 2.4. IEC Measurement

First, the membrane was cut into pieces weighing more than 0.025 g. The membrane was then placed in a vacuum oven at 80 °C for 24 h to remove water. After removal, the dehydrated dry membrane (W_dry,g_) was weighed. Subsequently, the dry membrane was immersed in a 1 M NaCl aqueous solution with a magnetic stir bar and stirred continuously for at least 24 h. This allowed the protons (H^+^) on the sulfonate groups within the membrane to be replaced by sodium ions (Na^+^) in the solution, forming NaSO_3_. Prior to measurement, the membrane was removed, and 3–5 drops of phenolphthalein were added as an indicator to the original NaCl solution in which the membrane had been soaked. The solution was then titrated with a prepared 0.01 M NaOH aqueous solution until neutralization was achieved, from which the consumed volume of the titration solution (V_NaOH_) was determined. Finally, the IEC value was calculated by substituting the variables into the following equation, as shown in Table 2 [14,15,16,17].

IEC Formula:

$$IEC\left(mmol/g\right)=\frac{\left(V_{NaOH},\;ml\right)\times \left(N_{NaOH},\;N\right)}{\left(W_{dry},g\right)}$$

where *V_NaOH_* = Volume of the NaOH solution (mL);

*N_NaOH_* = Normality (OH^−^ equivalent concentration) of the NaOH solution (determined by KHP standardization);

*W_dry_* = Weight of the dry membrane (g).

### 2.5. Physicochemical Stability and Water Uptake Properties

First, the membrane was cut into pieces of approximately 2 × 1 cm and dried in a vacuum oven at 70 °C for 12 h. After removal, the dry membrane (W_dry_) was weighed and subsequently immersed in a vial containing deionized (DI) water. The vials were maintained at 30, 50, 70, 80, and 90 °C. After soaking for 24 h, the membranes were removed. Excess water droplets on the membrane surface were carefully blotted using lint-free paper, followed immediately by weighing the wet membrane (W_wet_). Subsequently, the changes in the length, width, and thickness of the membranes were measured. The obtained results were then substituted into the corresponding equations to calculate each parameter [18,19,20].

$$Water\;uptake\left(\Delta wt\%\right)=\frac{W_{wet}-W_{dry}}{W_{dry}}\times 100\%$$

where *W_wet_* = Weight of the wet membrane (mg);

*W_dry_* = Weight of the dry membrane (mg);

$$Dimensional\;stability\left(\Delta L\%\right)=\frac{L_{wet}-L_{dry}}{L_{dry}}\times 100\%$$

where *L_wet_* = Dimension (length, width, or thickness) of the wet membrane (mm);

*L_dry_* = Dimension (length, width, or thickness) of the dry membrane (mm).

Hydration Number (λ)

The λ value is defined as the number of water molecules per sulfonate group.

It is calculated as follows:

$$\lambda =\frac{\left[H_{2}O\right]}{\left[{SO_{3}}^{-}\right]}=\frac{Water\;uptake\times 10}{M_{H_{2}O}\times IEC(mmol/g)}$$

where $M_{H_{2}O}$ = Molecular weight of water.

The membrane was cut into pieces measuring 2 × 1 cm and placed in a vacuum oven at 70 °C for dehydration. After removal, the dry membrane (W_dry_) was weighed. Subsequently, the membrane was immersed in Fenton’s reagent, which was prepared with 3% H_2_O_2_ and 2 ppm Fe_2_(SO_4_)_3_, and then placed in a convection oven at 80 °C for 1 h. After removal, the membrane surface was washed with DI water, and excess moisture was gently blotted dry using lint-free paper. The membrane was then placed back into the vacuum oven at 70 °C for dehydration. Finally, the membrane was removed, and the dry weight of the post-test membrane (W_dry-Fenton_) was recorded [21,22,23].

$$Stability\left(\Delta wt\%\right)=(1-\frac{W_{before}-W_{after}}{W_{after}})\times 100\%$$

## 3. Results

### 3.1. GPC Analysis

In this study, polyfluorinated and diol monomers were synthesized through a series of chemical reactions, after which three variations of alternating poly(arylene ether) polymer structures bearing polyfluoro substituents were successfully prepared via nucleophilic polycondensation. This series of polymers was subsequently analyzed using gel permeation chromatography (GPC).

As summarized in Table 3, the weight-average molecular weights (M_w_) of these polymers ranged between 42,000 and 180,000 g/mol, with the polydispersity index (PDI) falling within the range of 1.03 to 1.36. These PDI values indicate that the polymers prepared in this work exhibit narrow molecular weight distributions. This is presumably because the precursor monomers of this polymer series were all verified by high-performance liquid chromatography (HPLC) to have a purity of 100% prior to polymerization, which allowed the resulting polymers to achieve such high uniformity [24,25].

### 3.2. Fourier Transform Infrared (FT-IR) Spectroscopic Analysis

To confirm the polymer structures, the chemical structures of a series of polymers and sulfonated polymers were analyzed using FTIR spectroscopy. The analysis was conducted within a wavenumber measurement range of 650–4000 cm^−1^.

**Figure 11:**
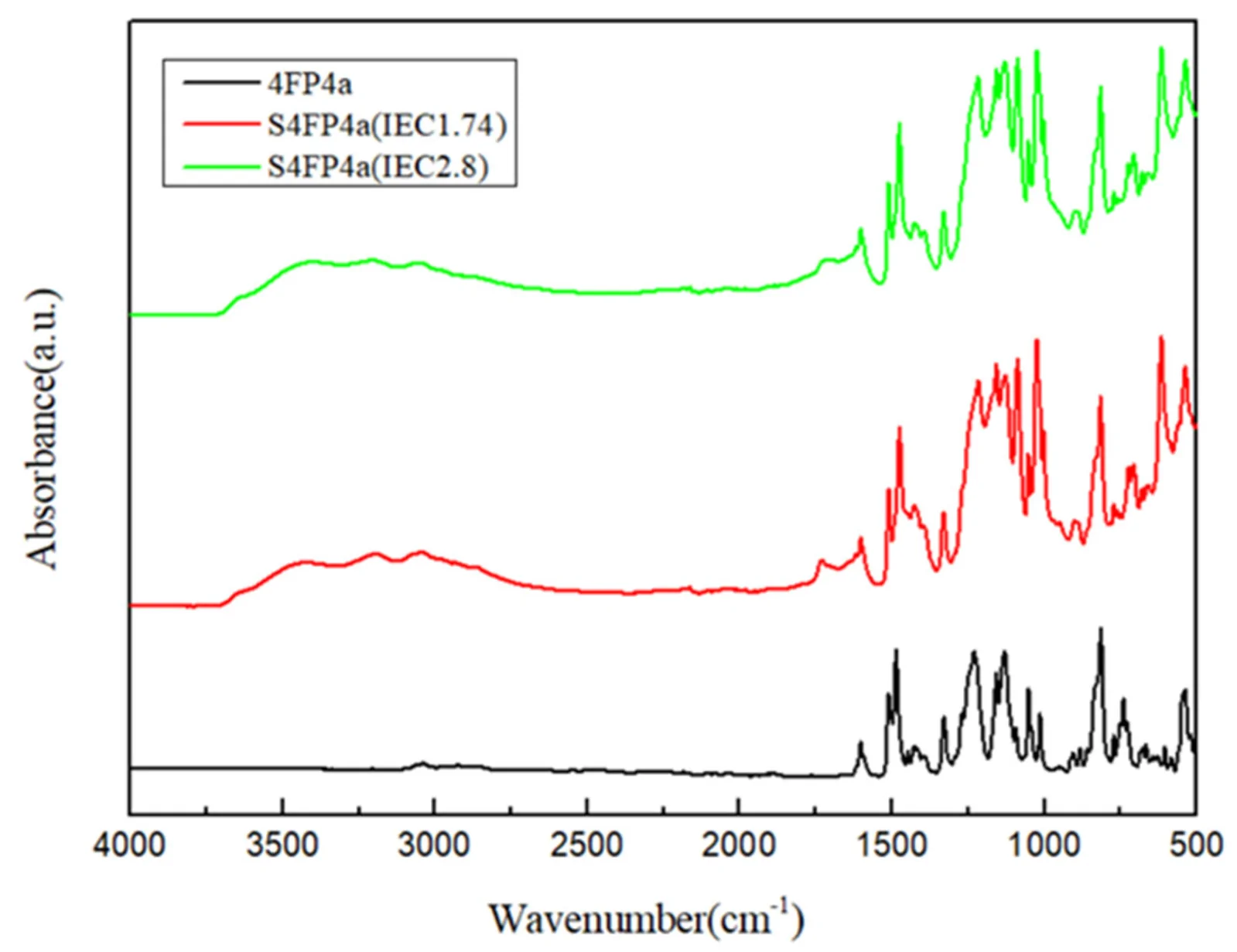
4FP4a series FTIR spectra (650–4000 cm^−1^).

**Figure 12:**
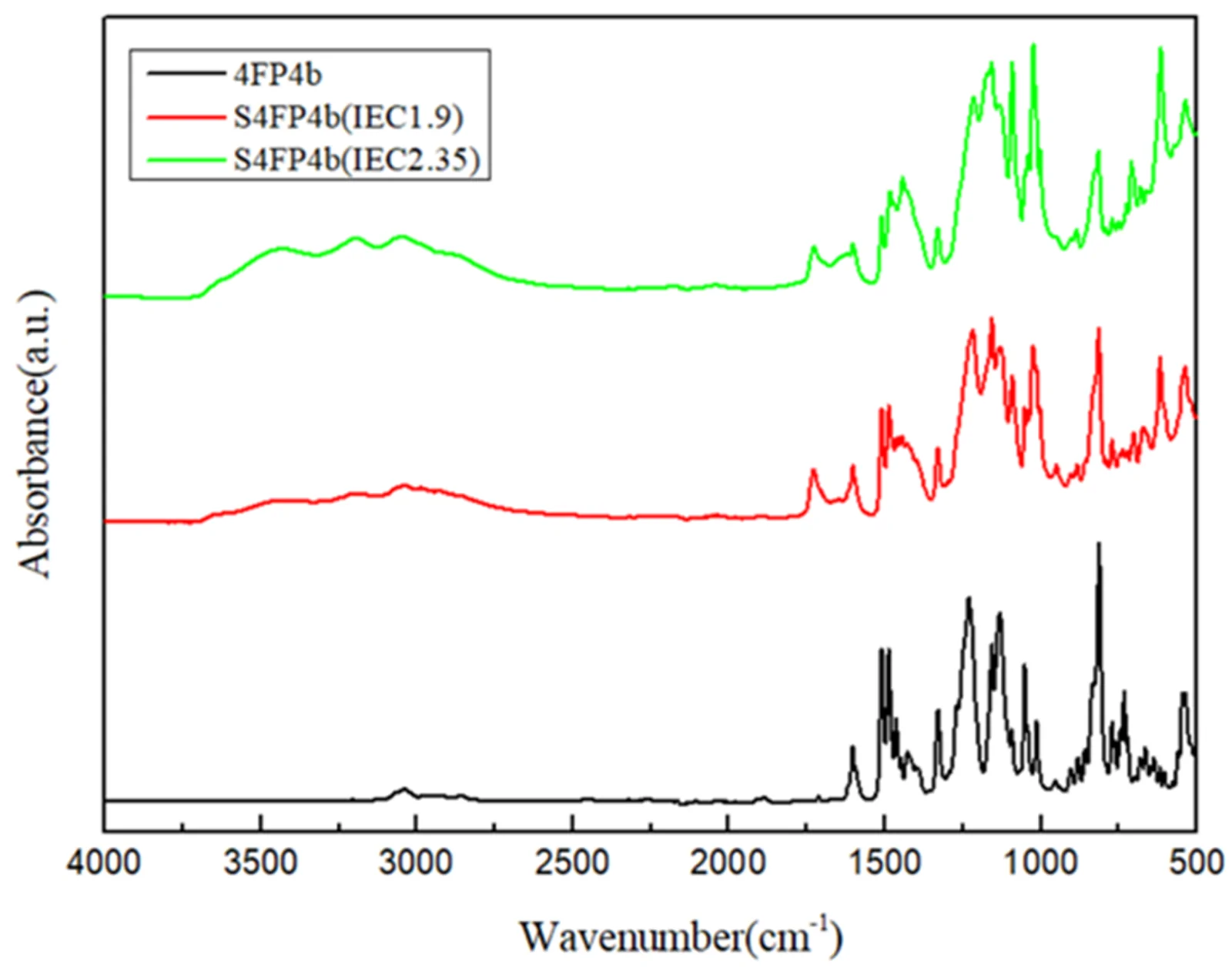
4FP4b series FTIR spectra (650–4000 cm^−1^).

**Figure 13:**
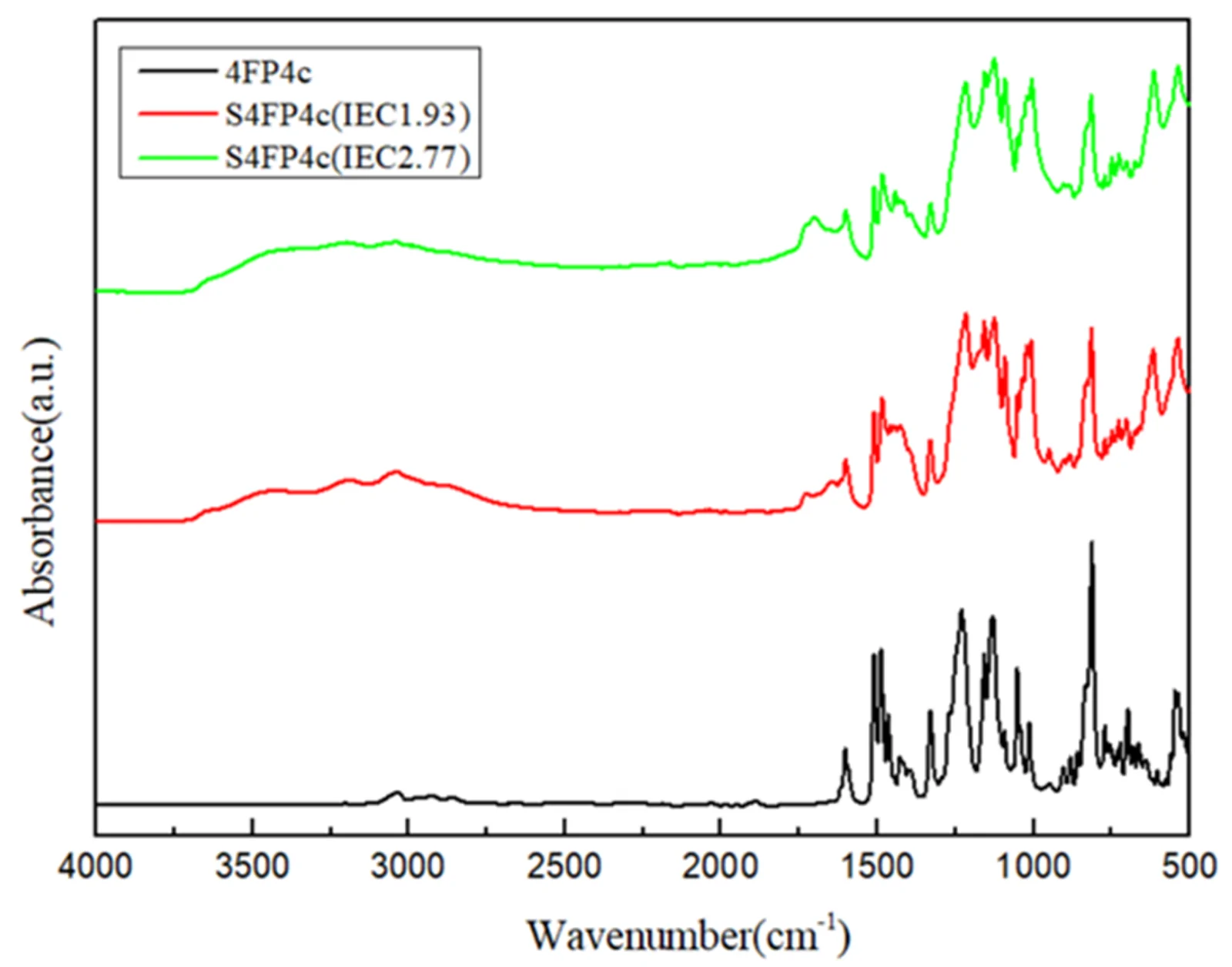
4FP4c series FT-IR spectra (650–4000 cm^−1^).

Figure 11, Figure 12 and Figure 13 show the FT-IR spectra of the 4FP4 and S4FP4 series. Because the sulfonated polymers (S4FP4 series) are grafted with hydrophilic sulfonate groups, they absorb moisture from the air; consequently, a characteristic hydroxyl (O-H) absorption peak emerges in the range of 3200–3500 cm^−1^. In contrast, no absorption peaks were observed in this region for the pristine polymers (4FP4 series) due to the absence of hydrophilic sulfonate groups [26,27,28].

**Figure 14:**
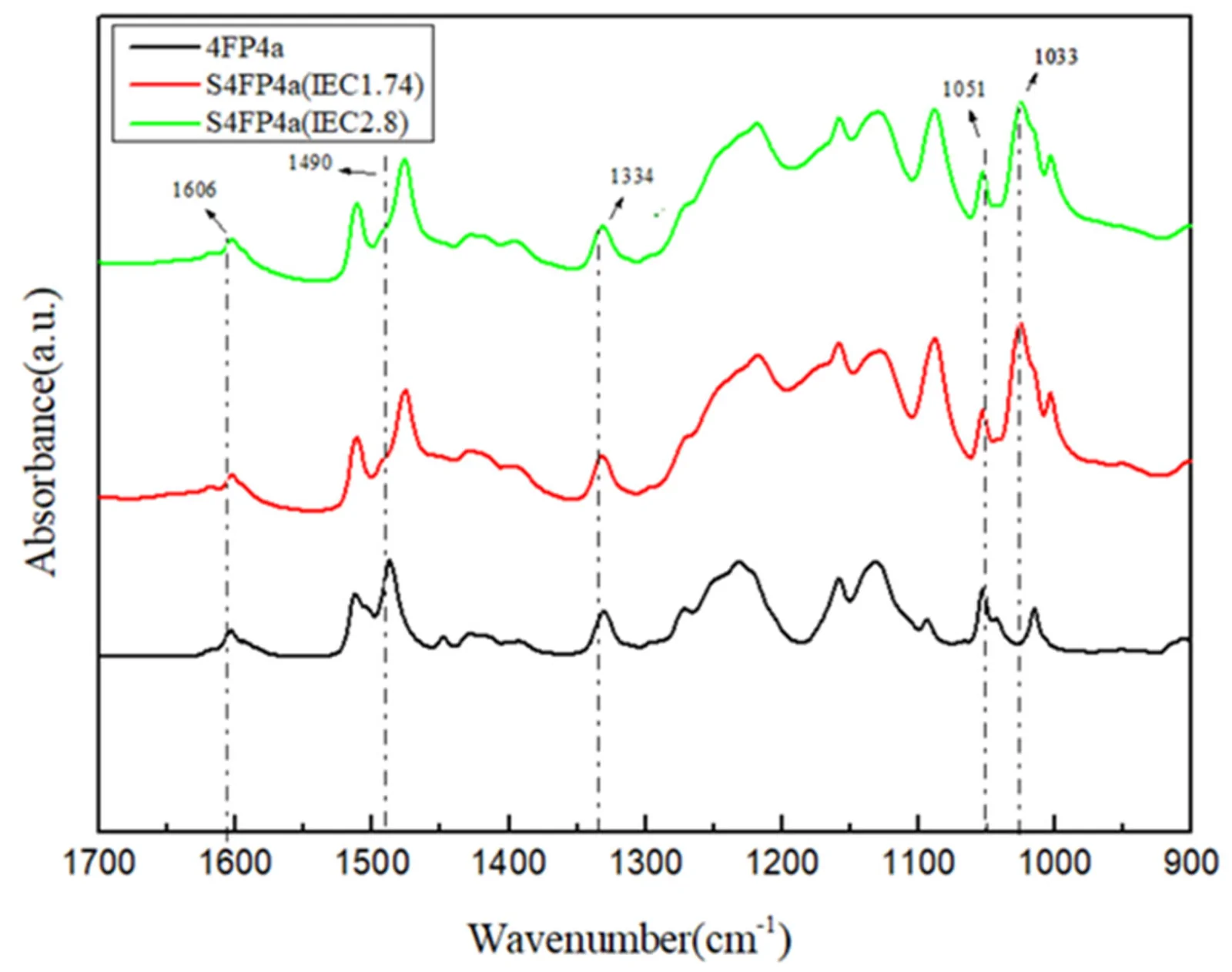
4FP4a series FT-IR spectra (900–1700 cm^−1^).

**Figure 15:**
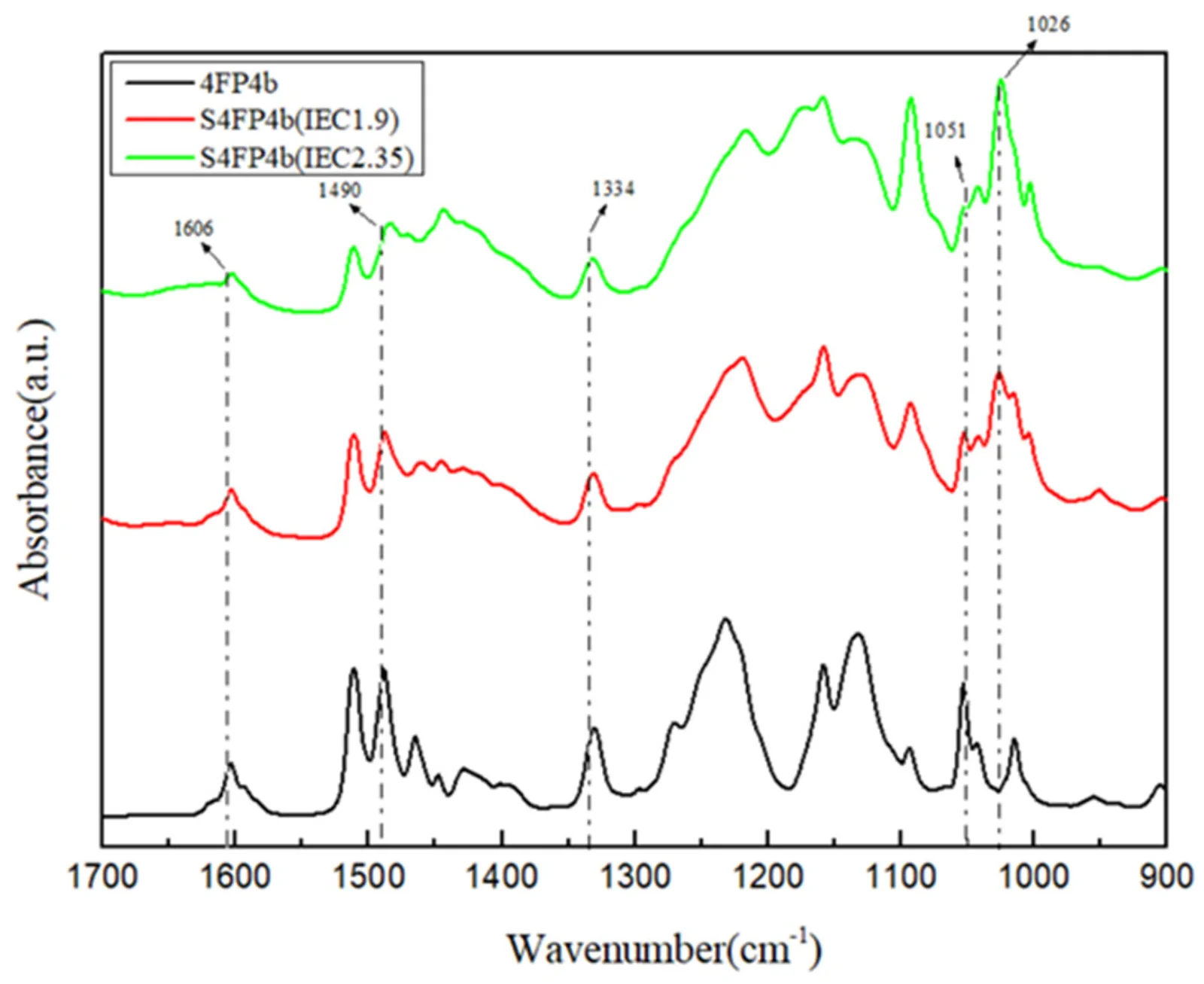
4FP4b series FT-IR spectra (900–1700 cm^−1^).

**Figure 16:**
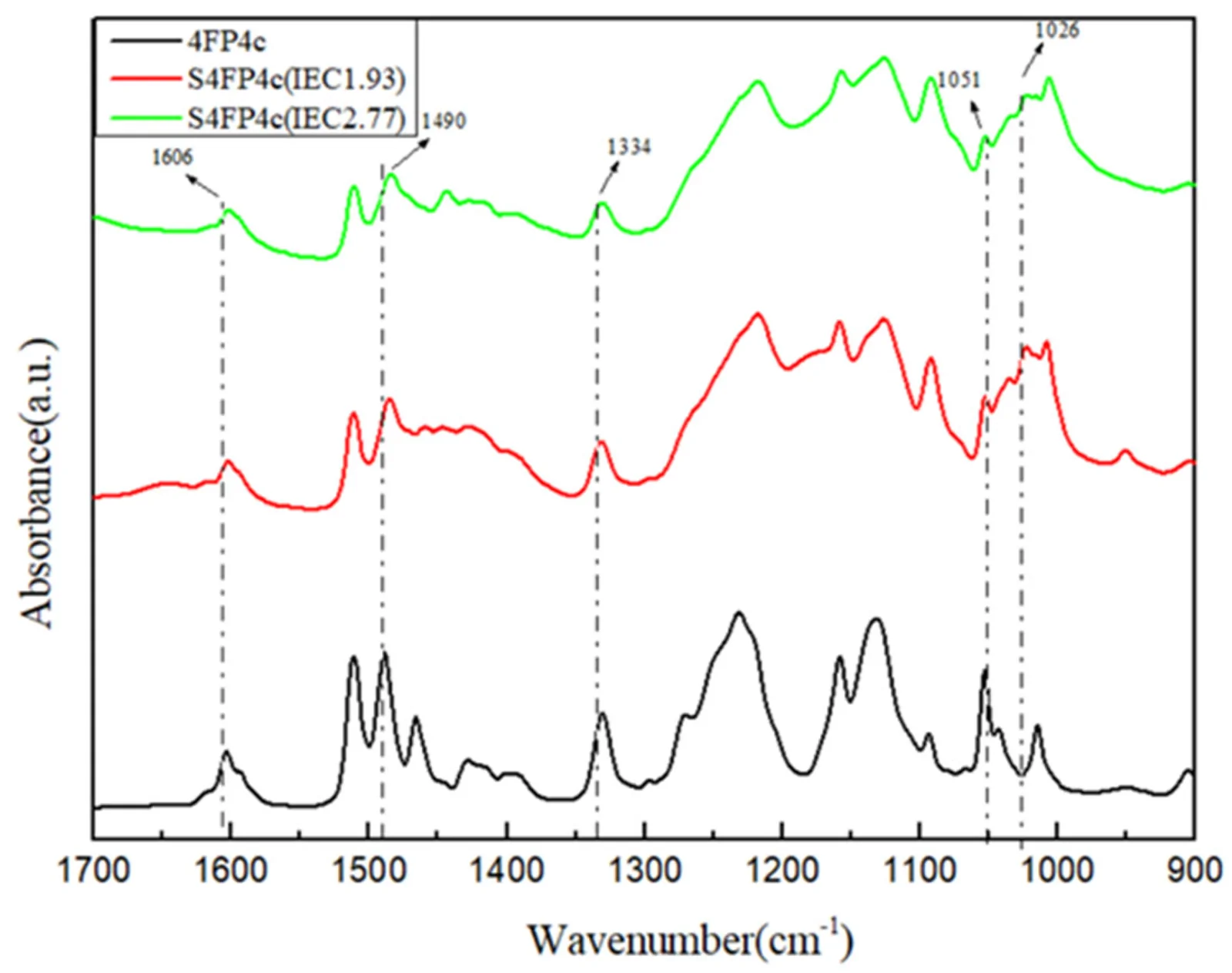
4FP4c series FT-IR spectra (900–1700 cm^−1^).

To further characterize the chemical structures of the polymers, the analysis focused on the spectral range of 900–1700 cm^−1^, as shown in Figure 14, Figure 15 and Figure 16. Following sulfonation, characteristic symmetric stretching absorption peaks of the sulfonate groups (O=S=O) appear at 1026 and 1033 cm^−1^, confirming the successful grafting of sulfonate groups onto the benzene ring structure of the polymers. The sulfonated polymers exhibit distinct peaks at these wavenumbers compared to the unsulfonated polymers; generally, the intensity of this absorption peak increases with a higher degree of sulfonation. Additionally, characteristic symmetric and asymmetric stretching absorption peaks of the ether groups (Ph-O-Ph) appear at 1051, 1334, and 1490 cm^−1^, respectively. Absorption peaks corresponding to the stretching vibrations of C-F bonds emerge within the range 1127–1240 cm^−1^. Finally, a characteristic absorption peak associated with the C=C stretching vibration of the aromatic rings is located at 1606 cm^−1^. The chemical structures of both the 4FP4 series and S4FP4 series were confirmed [29,30]. The spectra shown in Figure 11, Figure 12, Figure 13, Figure 14, Figure 15 and Figure 16 were acquired using attenuated total reflectance Fourier-transform infrared spectroscopy (ATR-FTIR) rather than conventional transmission FT-IR. In ATR measurements, polymer membranes can be analyzed directly in the solid state without preparing transparent transmission films or KBr pellets, and the instrument/software can report the spectra in absorbance units.

The chemical structures of the pristine and sulfonated polymers were characterized by attenuated total reflectance Fourier-transform infrared spectroscopy (ATR-FTIR) over the range of 4000–650 cm^−1^. The spectra are presented in absorbance units as generated by the FT-IR acquisition software Thermo Scientific Nicolet (OMNIC). The purpose of Figure 11, Figure 12, Figure 13, Figure 14, Figure 15 and Figure 16 is to compare the characteristic bands of the pristine and sulfonated polymers, particularly the O=S=O, Ph–O–Ph, C–F, and aromatic C=C stretching bands.

### 3.3. TGA Thermal Stability Analysis

**Figure 17:**
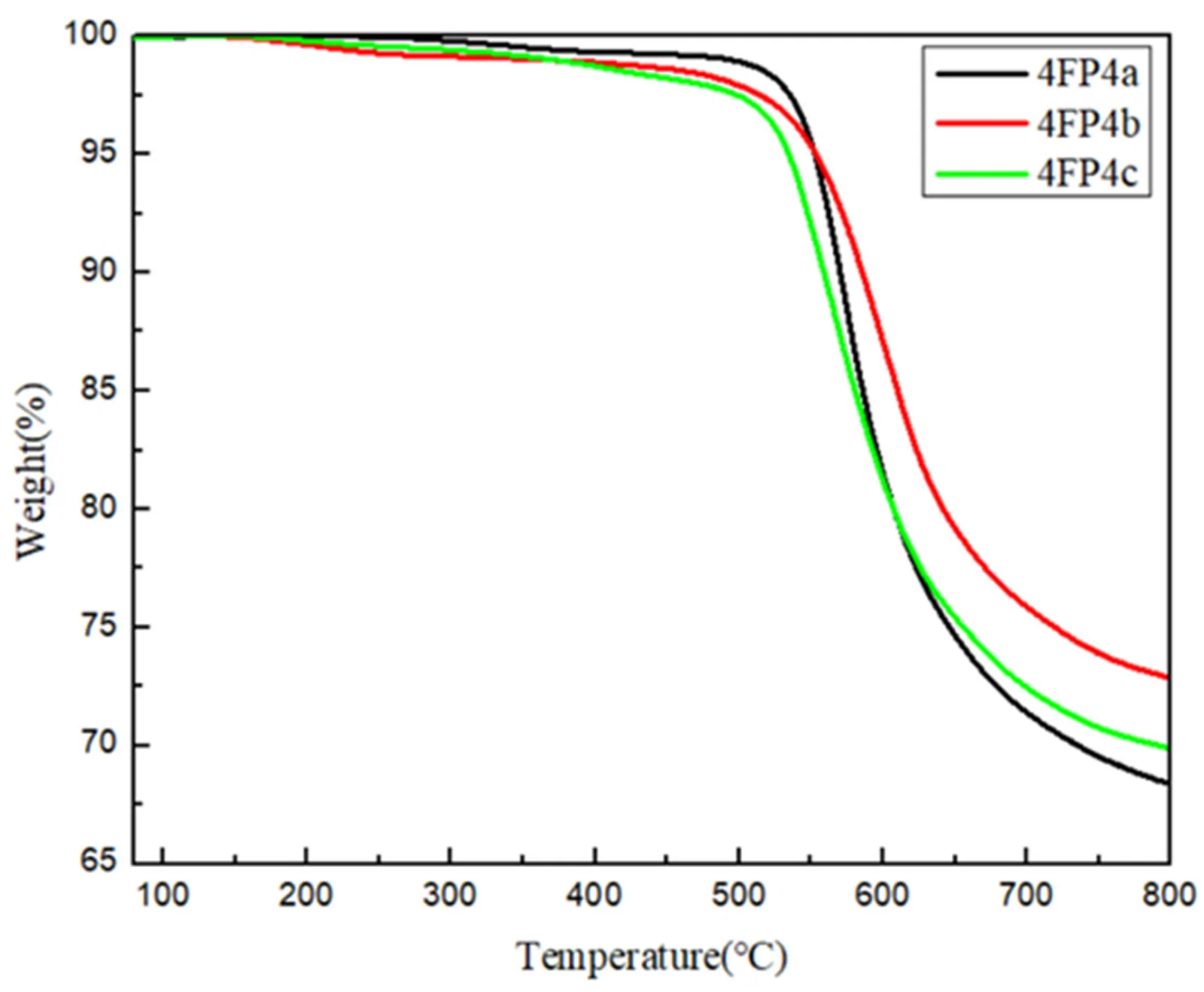
TGA curves of the polymers.

Generally, the thermal stability of a material is evaluated based on its thermal decomposition temperature (T_d5%_), which is defined as the temperature at which the material exhibits a 5% weight loss. Figure 17 shows the weight loss behavior of the unsulfonated polymers (4FP4 series) as a function of increasing temperature, and their corresponding thermal decomposition temperatures (T_d5%_) are summarized in Table 4. Due to the rigid nature of the multi-phenyl ring structures and the fluorenyl groups within the polymer backbone, the thermal decomposition temperatures of this series of polymers all exceed 530 °C, with 4FP4a and 4FP4b even reaching above 550 °C. Furthermore, all three polymers retained more than 65% of their residual weight at 800 °C, demonstrating excellent thermal stability. A closer investigation reveals that $4FP4c\;\left(M_{w}\approx 42\;\mathrm{kDa}\right)$, which possesses a lower molecular weight, exhibits slightly inferior thermal stability compared to the other two higher-molecular-weight polymers. Consequently, a correlation exists between the thermal decomposition temperature of a polymer and its molecular weight, where T_d5%_ tends to rise with an increase in the molecular weight of the polymer.

**Figure 18:**
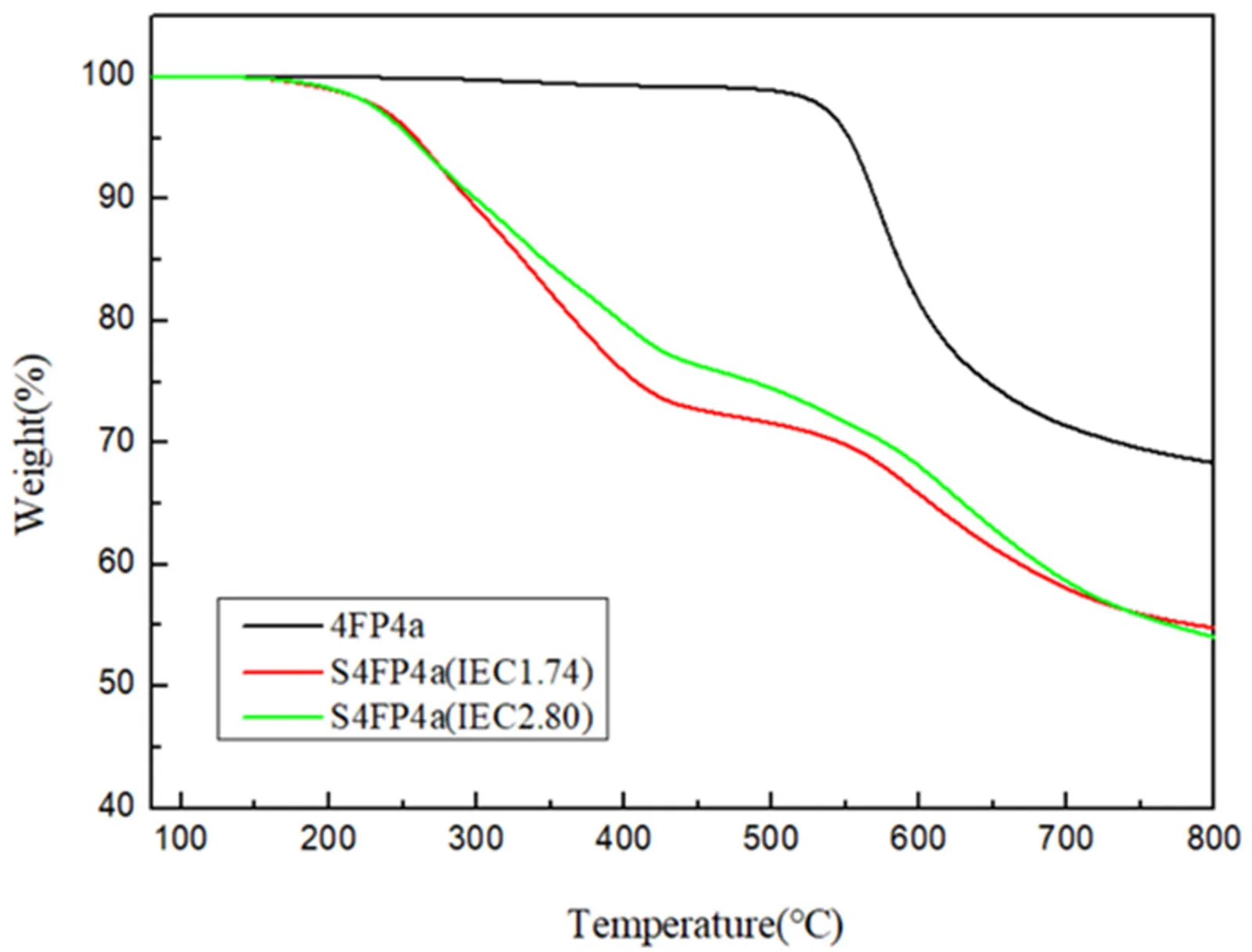
TGA curves of the S4FP4a series.

**Figure 19:**
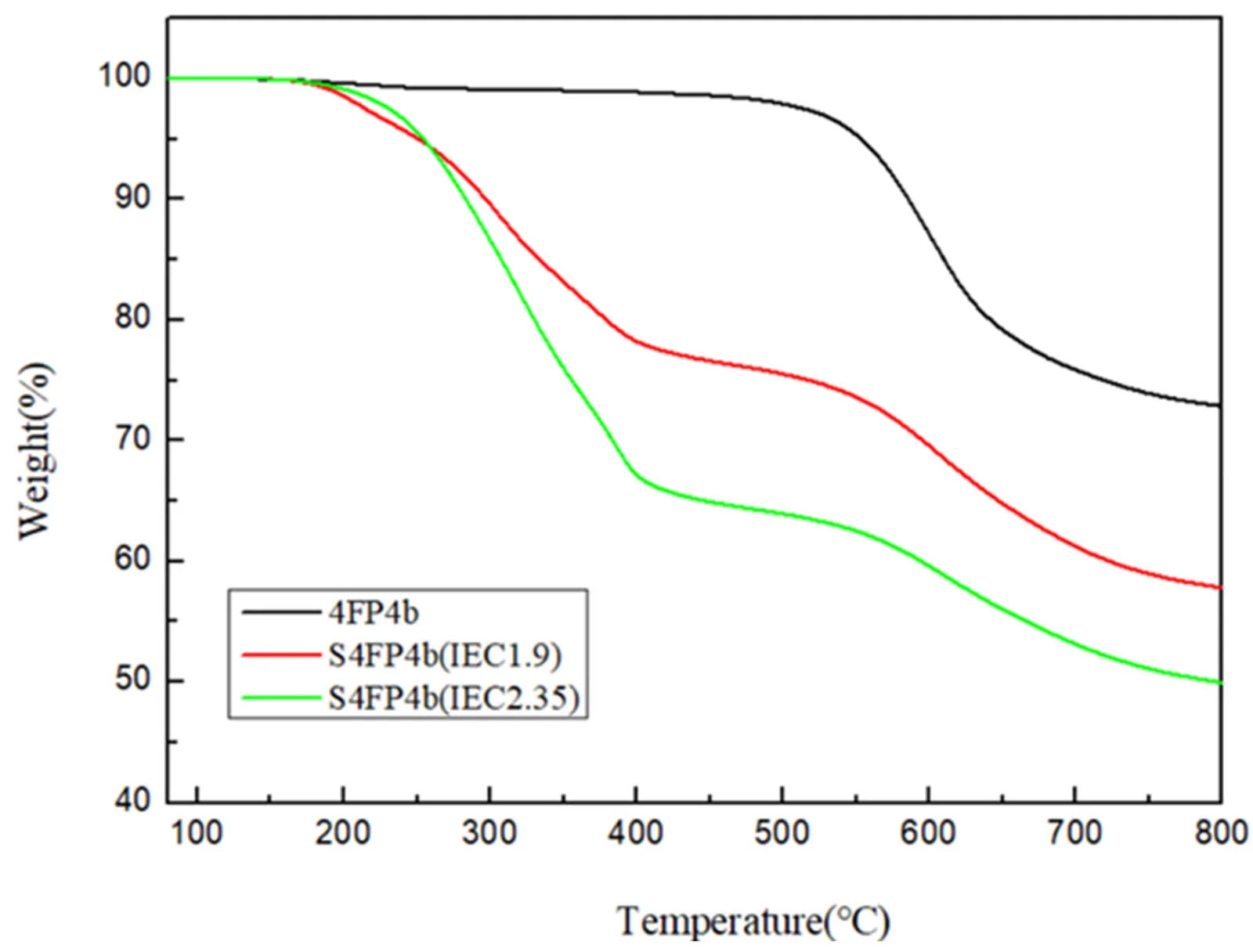
TGA curves of the S4FP4b series.

**Figure 20:**
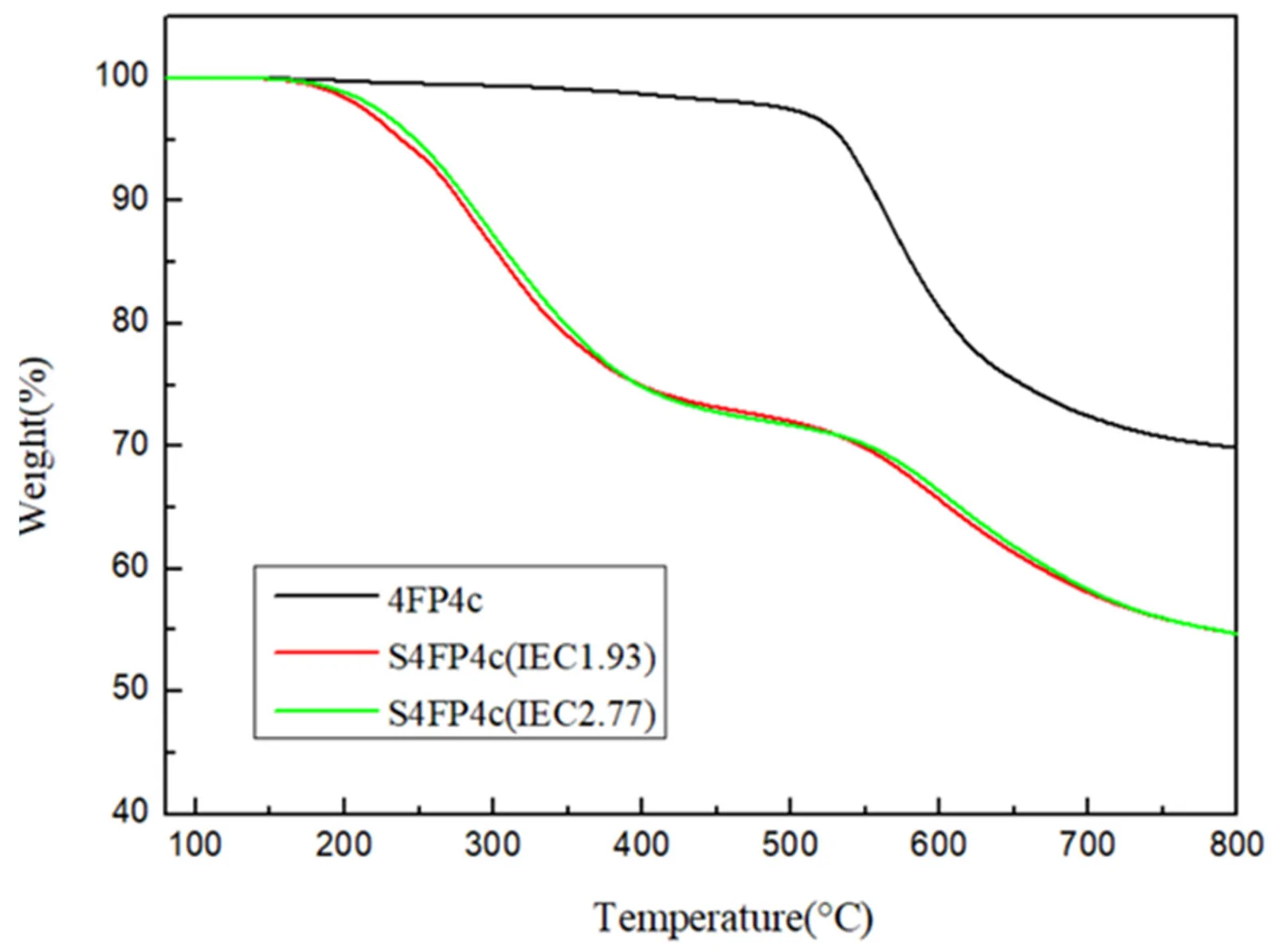
TGA curves of the S4FP4c series.

Figure 18, Figure 19 and Figure 20 display the TGA curves of the sulfonated polymers (S4FP4 series) analyzed within a temperature range of 80–800 °C. To prevent excessive moisture content in the membranes due to the hydrophilic nature of the abundant sulfonate groups, which could distort the thermogravimetric weight loss data, the sulfonated polymers were preheated to 120 °C and held for 30 min under a nitrogen atmosphere for dehydration before being cooled to 80 °C. Subsequently, the temperature was raised from 80 to 800 °C at a heating rate of 10 °C/min to enhance the accuracy of the measurement data.

The weight loss curves of the sulfonated polymers exhibit a distinct two-stage thermal decomposition behavior, and their corresponding thermal decomposition temperatures (T_d5%_) are summarized in Table 5. The first decomposition stage occurs between approximately 200 and 500 °C, which is primarily attributed to the degradation of the sulfonate groups. Generally, this weight loss becomes more pronounced with an increasing degree of sulfonation. The second stage corresponds to the degradation of the polymer backbone, which predominantly occurs above 500 °C. These data demonstrate that the multi-phenyl ring and fluorenyl group structures within the polymer chains impart excellent thermal stability to the 4FP4 series. Moreover, the thermal decomposition temperatures (T_d5%_) of the S4FP4 series are all above 238 °C, with that of S4FP4a (IEC 1.74) reaching as high as 259 °C. Furthermore, all polymers in this series retain more than 50% of their residual weight at 800 °C, demonstrating exceptionally outstanding thermal stability [31,32].

### 3.4. Physicochemical Stability and Water Uptake Properties of Membranes

Because proton transport is mediated by water molecules, a favorable water uptake directly enhances the proton conductivity of the membranes. However, excessive water swelling can compromise their dimensional stability, potentially leading to structural deformation or dissolution in water. Therefore, maintaining an optimal water uptake is essential for the membranes to balance high proton conductivity with excellent dimensional stability. To evaluate water uptake and dimensional stability, the membrane was first cut into pieces measuring 2 × 1 cm and dried in a vacuum oven for 24 h. After recording the dry weight and initial dimensions, the membrane was immersed in DI water and maintained at various temperatures (30, 50, 70, 80, and 90 °C) for 24 h. Subsequently, the membrane was removed to record its wet weight and dimensions, allowing for the comprehensive tracking of water uptake behavior and dimensional changes as a function of temperature. These results are compiled in Table 6.

**Figure 21:**
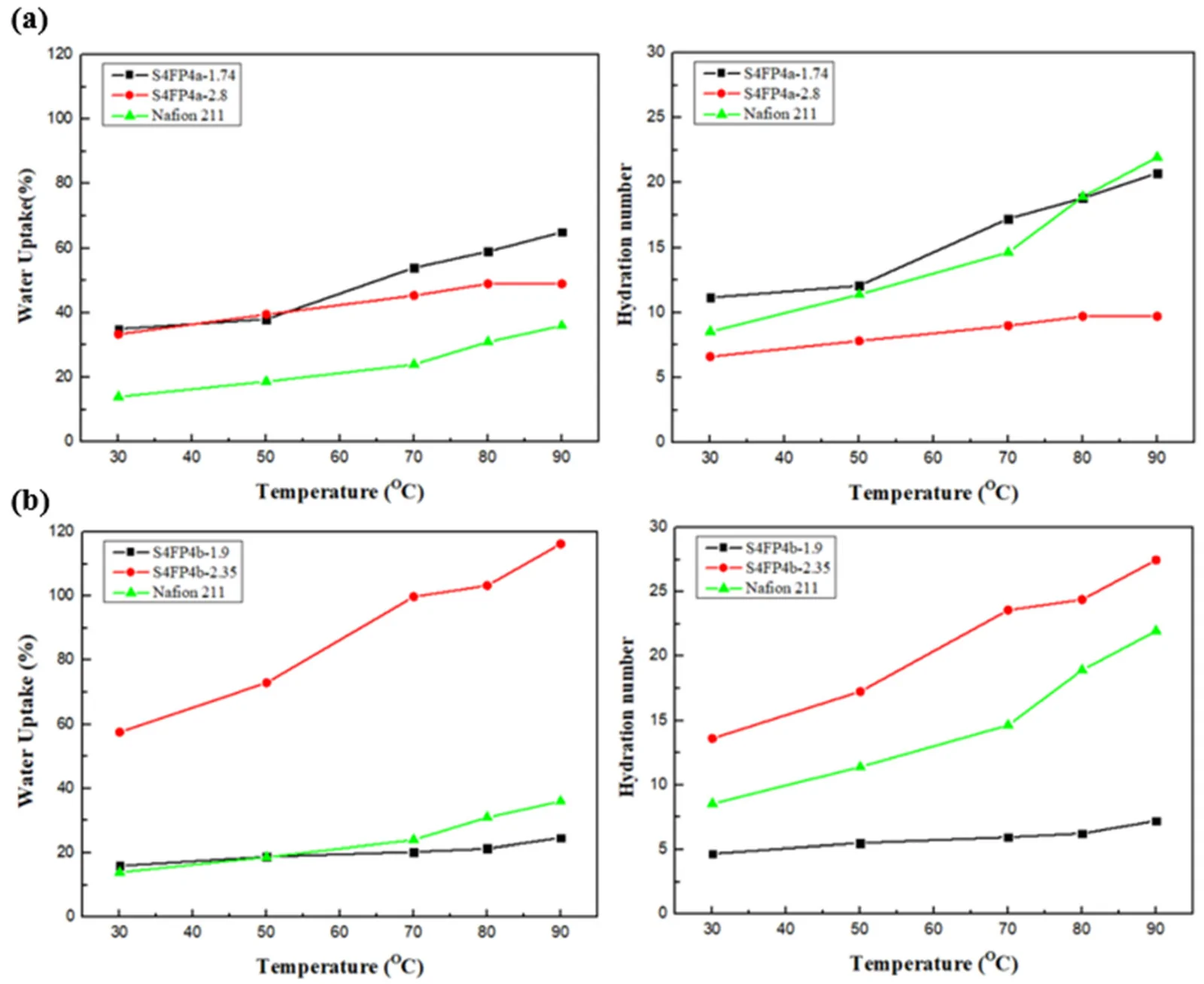

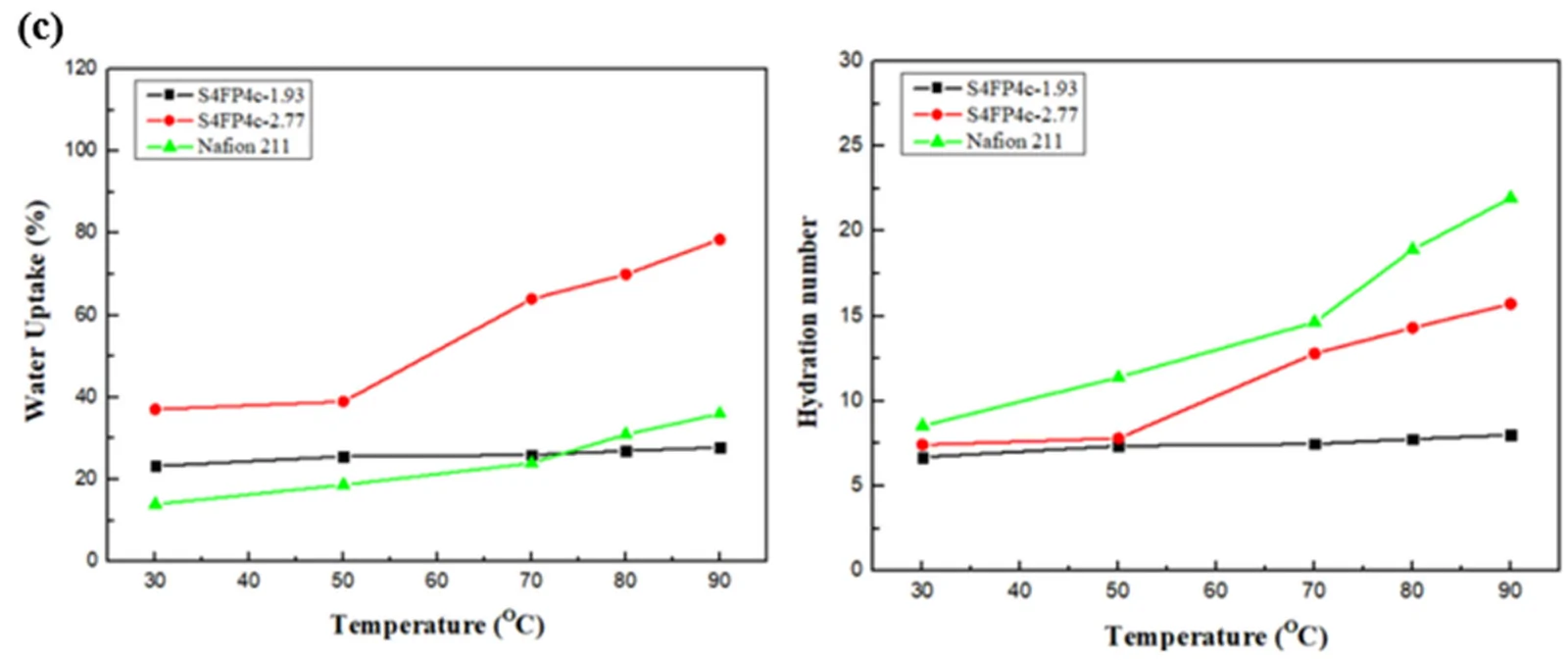
Water uptake and λ values of the sulfonated polymers at various temperatures: (**a**) S4FP4a, (**b**) S4FP4b, and (**c**) S4FP4c.

Figure 21a–c show the temperature-dependent water uptake and hydration numbers (λ) of the sulfonated polymers (S4FP4 series). Crucially, all S4FP4 series membranes maintained their structural integrity even at 90 °C, demonstrating robust dimensional stability. Within the temperature range of 30–90 °C, the water uptake of all sulfonated polymers ranged from 16% to 116.3%. As shown in Figure 21a, both S4FP4a (IEC 1.74) and S4FP4a (IEC 2.80) exhibited water uptake values exceeding 30% at 30 °C, which gradually increased with rising temperature. At 90 °C, the water uptake of S4FP4a (IEC 1.74) and S4FP4a (IEC 2.80) increased to 65% and 49%, respectively. As observed in Figure 21b, S4FP4b (IEC 1.90) displayed a water uptake behavior similar to that of Nafion 211, with values falling within the range of 16–24.7% between 30 and 90 °C. Generally, the water uptake expanded as the IEC of the sulfonated polymers increased; S4FP4b (IEC 2.35) showed a water uptake surpassing 50% at 30 °C, which escalated to 116.3% when the temperature reached 90 °C. Meanwhile, Figure 21c demonstrates that S4FP4c (IEC 1.93) exhibited low temperature dependence, with its water uptake hovering between 23% and 27% from 30 to 90 °C. In contrast, the water uptake of S4FP4c (IEC 2.77) displayed an upward trend, increasing from 37% to 78% with rising temperature.

Typically, the water uptake of a membrane is positively correlated with the IEC value of the polymer; a higher IEC value generally indicates a higher degree of sulfonation, meaning a larger number of sulfonate groups within the membrane matrix, which in turn enhances its water retention capacity. However, as listed in Table 6, the relationship between the IEC value and water uptake of S4FP4a exhibited a distinct trend compared to the other two materials. Specifically, S4FP4a (IEC 1.74), despite its lower IEC, showed a higher water uptake than S4FP4a (IEC 2.80). This anomaly is attributed to the non-uniform distribution of sulfonate groups within the membrane matrix (i.e., excessive local concentration of sulfonate groups), which led to regional variations in the hydration capacity of the sulfonate groups, inducing the aforementioned behavior. Regarding the dimensional stability, all S4FP4 series membranes displayed a dimensional change ratio ranging from 2% to 21% at 30 °C. As the temperature rose to 90 °C, all S4FP4 series membranes successfully maintained favorable dimensional change ratios (2.5–26.3%). Taking the S4FP4a series as an example, it preserved an exceptional dimensional change ratio (11–17%) even at an elevated temperature of 90 °C, significantly outperforming the previously developed SP4 series (30–70%).

The hydration number (λ, nH_2_O/nSO_3_H) of a sulfonated polymer represents the molar ratio of water molecules to sulfonate groups inside the membrane matrix; a higher λ value signifies a more densely hydrated environment surrounding each sulfonate group. As shown in Figure 21, the λ values of the high-IEC S4FP4a (IEC 2.80), low-IEC S4FP4b (IEC 1.90), and S4FP4c-1.93 did not increase significantly with rising temperature. These three materials exhibited low temperature dependence. Conversely, S4FP4a (IEC 1.74) and S4FP4b (IEC 2.35) displayed λ values of 11.1 and 13.6 at 30 °C, respectively, both surpassing that of Nafion 211 (λ = 9). Even at 90 °C, they maintained λ values comparable to Nafion 211, demonstrating the excellent water retention capability of the sulfonate groups. In summary, regarding physical properties, introducing fluorine substituents onto the difluoro monomers of the sulfonated polymers (S4FP4 series) prepared in this work effectively regulated the grafting of sulfonate groups onto the diol monomers. Consequently, the S4FP4 series membranes substantially enhanced their dimensional stability while maintaining an appropriate level of water uptake [33,34,35].

During fuel cell operation, the cathode reduction process combines oxygen with protons and electrons to generate water. However, incomplete reduction can lead to the generation of hydrogen peroxide (H_2_O_2_), which may subsequently react with metal cations to form hydroxyl radicals (⋅OH). These radicals can decompose the polymer electrolyte within the fuel cell. Therefore, this study evaluates the oxidative and hydrolytic stabilities of the modified S4FP4 series membranes under highly oxidative and high-temperature environments.

Before the oxidative stability measurement, the membrane was placed in a vacuum oven for 12 h to dehydrate, and its dry weight was recorded upon removal. The membrane was then immersed in a vial containing a 2 ppm Fenton’s reagent solution and placed in an oven at 80 °C. After 1 h, the membrane was isolated, dried, and weighed to evaluate the weight-loss behavior. These results are summarized in Table 7. After exposure to Fenton’s reagent at 80 °C for 1 h, the S4FP4 membranes retained 96.4–100% of their initial dry mass, while Nafion 211 retained 98.0%. These results indicate that the S4FP4 membranes exhibit favorable short-term oxidative resistance comparable to Nafion 211 under the present test conditions. It should be emphasized that the present 1 h Fenton test was intended as a preliminary comparative screening rather than a long-term durability assessment. Extended Fenton exposure is required to establish the long-term oxidative durability of these membranes.

For the hydrolytic stability measurement, the membrane was first placed in a vacuum oven to dehydrate and weighed upon removal to obtain the dry membrane weight. Subsequently, the membrane was immersed in water, placed in a sand bath, and heated on a hot plate with the internal temperature regulated at 100 °C. After 24 h, the membrane was removed, gently blotted dry, and dried again to record the weight loss behavior. The measurement data are summarized in Table 8. Regarding the hydrolytic stability, the previously developed SP4 series exhibited a sharp decline in stability or even dissolved in water when the IEC exceeded 2.0 mmol/g. In contrast, the S4FP4 series prepared in this study retained more than 99% of its initial weight even after being placed in water at 100 °C for 24 h, demonstrating exceptionally outstanding hydrolytic stability.

## 4. Discussion

### 4.1. Proton Conductivity Data

**Figure 22:**
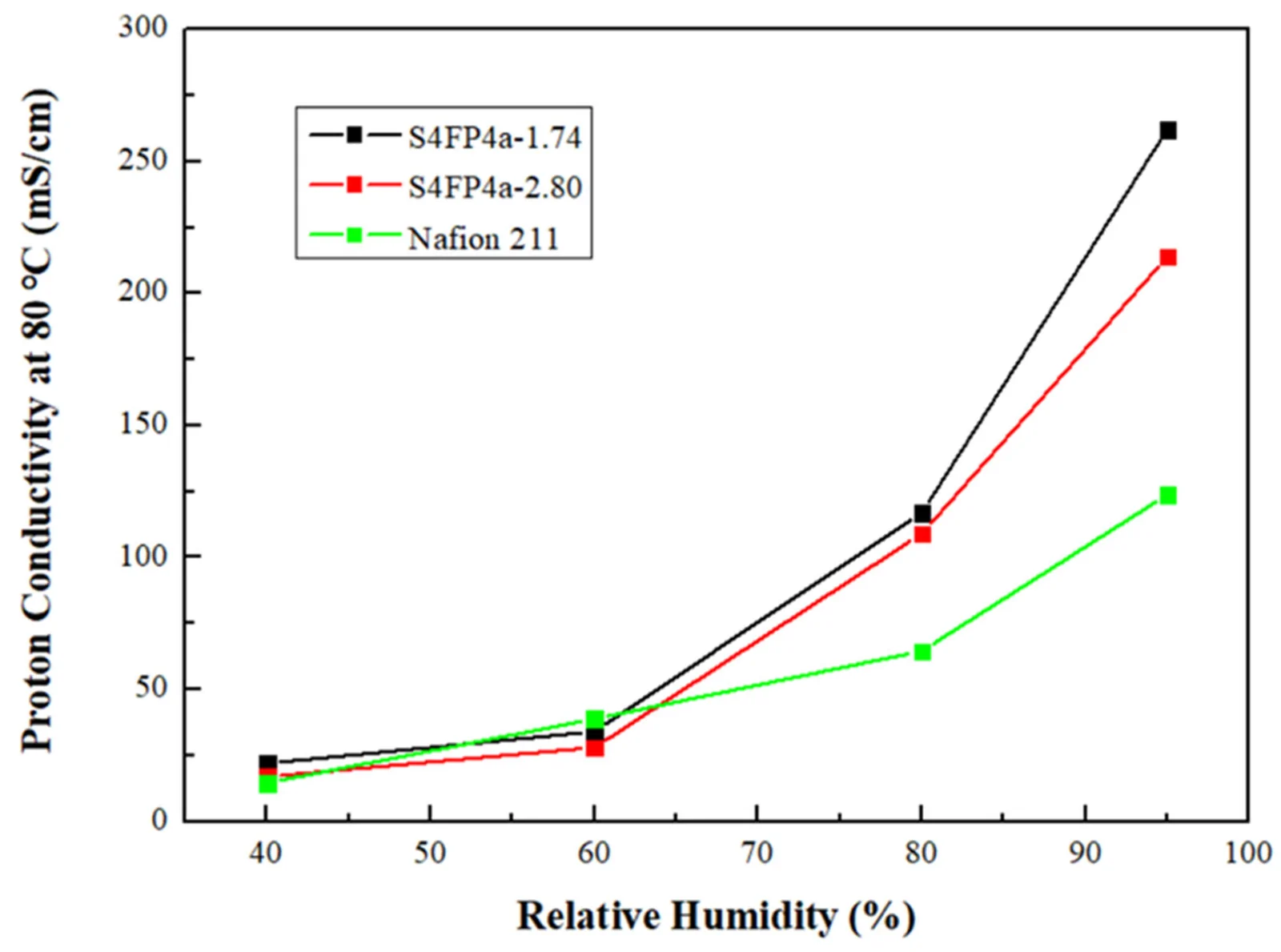
Proton conductivity of S4FP4a series at a fixed temperature of 80 °C under various humidities.

**Figure 23:**
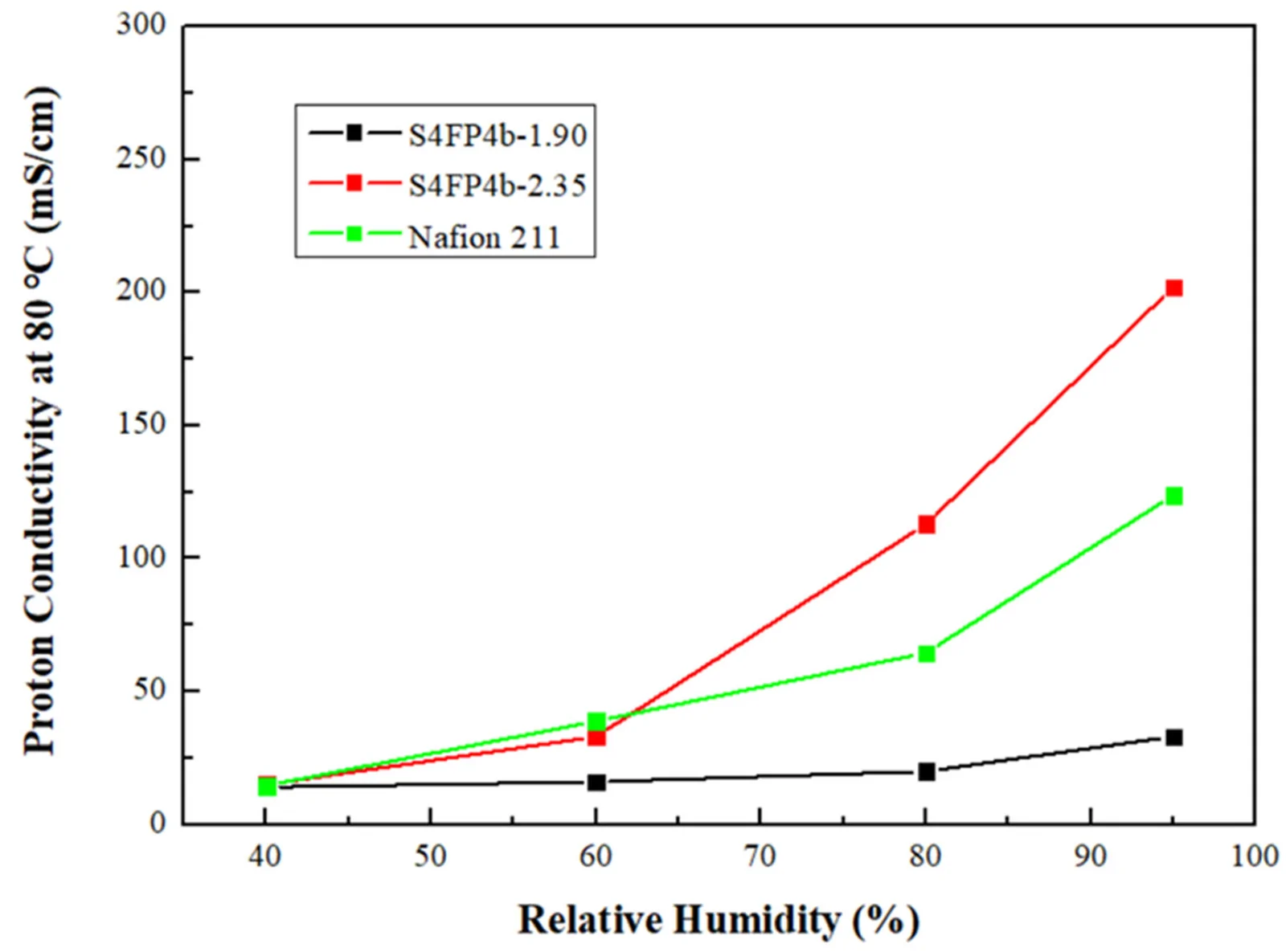
Proton conductivity of S4FP4b series membranes at a fixed temperature of 80 °C under various humidities.

**Figure 24:**
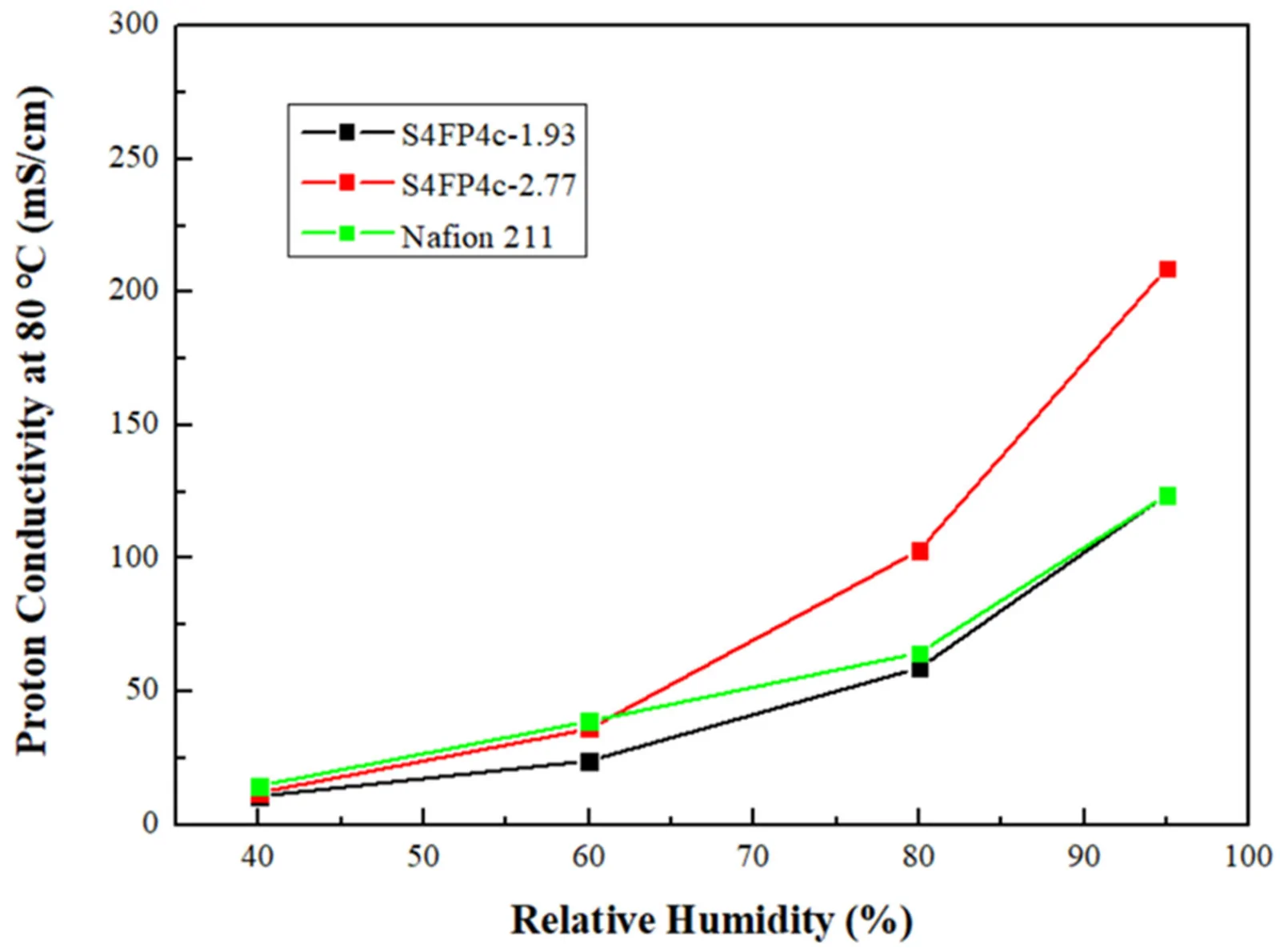
Proton conductivity of S4FP4c series membranes at a fixed temperature of 80 °C under various humidities.

The proton conductivity of the S4FP4 series membranes was determined by measuring the impedance values of each membrane at a fixed temperature of 80 °C under various relative humidities using an AC impedance analyzer. Figure 22, Figure 23 and Figure 24 illustrate the trends in proton conductivity for the S4FP4 series and Nafion 211 at 80 °C across different relative humidities. The results are summarized in Table 9. As shown in Table 9, in a low-humidity environment of 40% RH, the proton conductivity of the S4FP4 series ranges between 11 and 22 mS/cm, close to the 14.6 mS/cm of Nafion 211. In contrast, under a high-humidity environment of 95% RH, the proton conductivity of each S4FP4 series membrane ranges from 33 to 262 mS/cm. As shown in Figure 22, at 80 °C and 95% RH, the proton conductivities of S4FP4a (IEC 1.74) and S4FP4a (IEC 2.80) are 262 and 214 mS/cm, respectively; however, S4FP4a (IEC 1.74) maintains excellent dimensional stability even at an elevated temperature of 90 °C, with a dimensional change rate of only 17%. Furthermore, Figure 23 shows that the proton conductivity of S4FP4b (IEC 2.35) can reach as high as 202 mS/cm at 80 °C and 95% RH. As shown in Figure 24, the proton conductivities of S4FP4c (IEC 1.93) and S4FP4c (IEC 2.77) at 80 °C and 95% RH are 124 and 209 mS/cm, respectively.

These findings demonstrate that introducing fluorine can effectively enhance the proton conductivity of the membranes while maintaining favorable dimensional stability. Among them, the S4FP4a series exhibits relatively superior proton conductivity and dimensional stability, which is expected to yield promising performance in fuel cell applications. Additionally, despite having a lower IEC value, S4FP4a (IEC 1.74) achieves a higher proton conductivity than S4FP4a (IEC 2.80). This is because the proton channels within the S4FP4a (IEC 1.74) membrane are more uniformly distributed and appropriately spaced, resulting in a superior microphase-separated morphology.

### 4.2. TEM Microphase Separation Morphology

**Figure 25:**
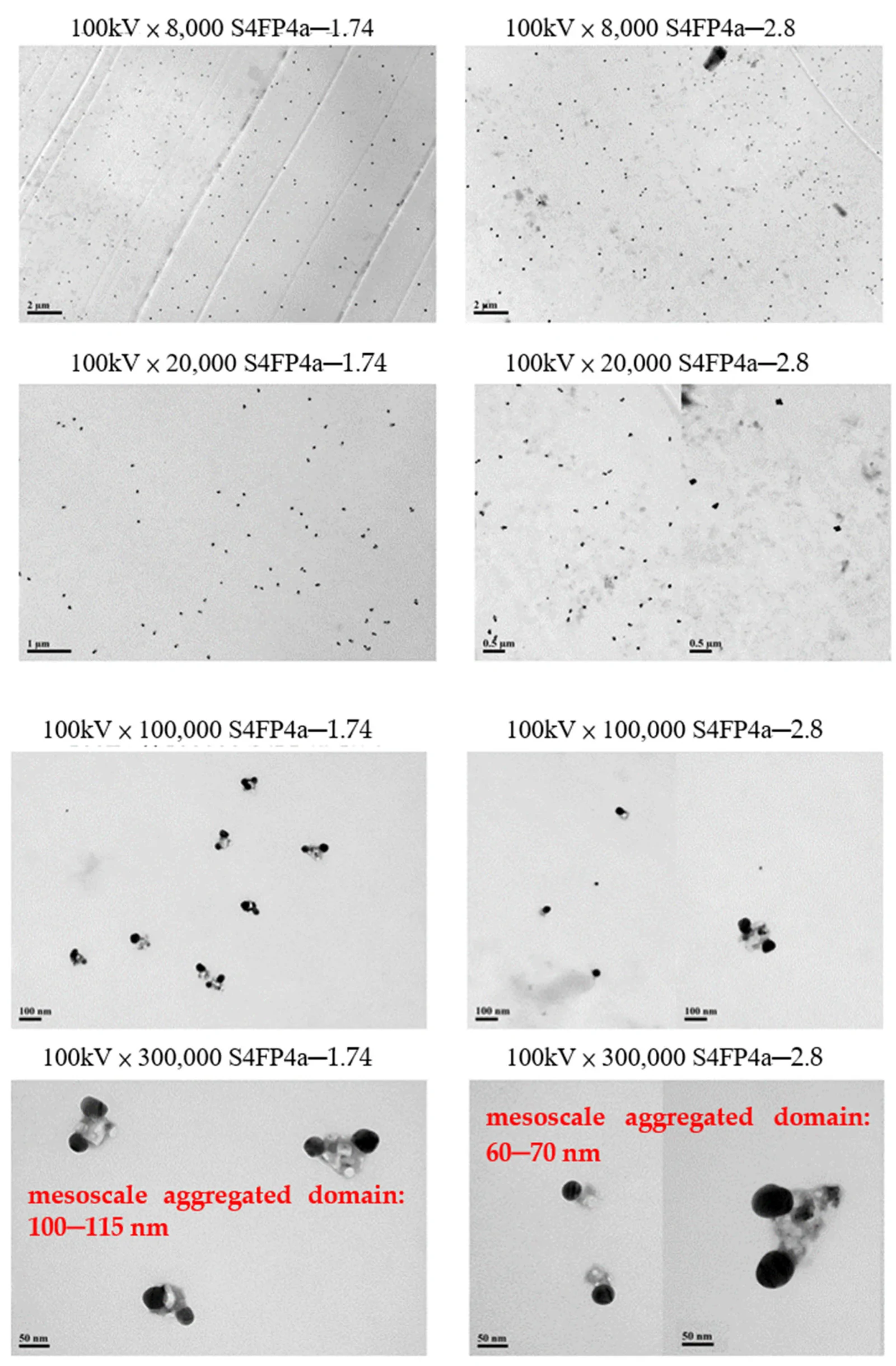
TEM images of S4FP4a series sulfonated polymers.

**Figure 26:**
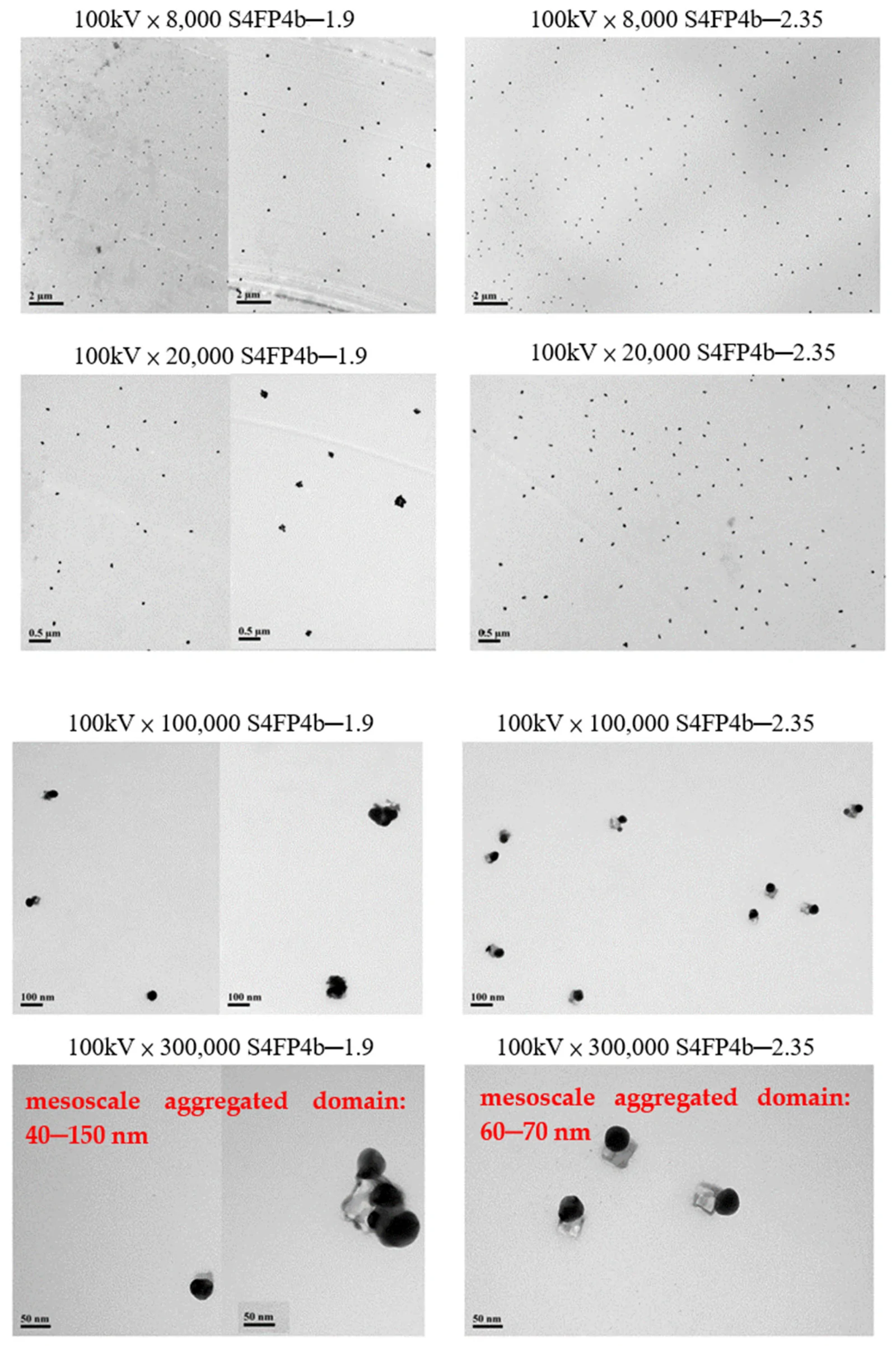
TEM images of S4FP4b series sulfonated polymers.

**Figure 27:**
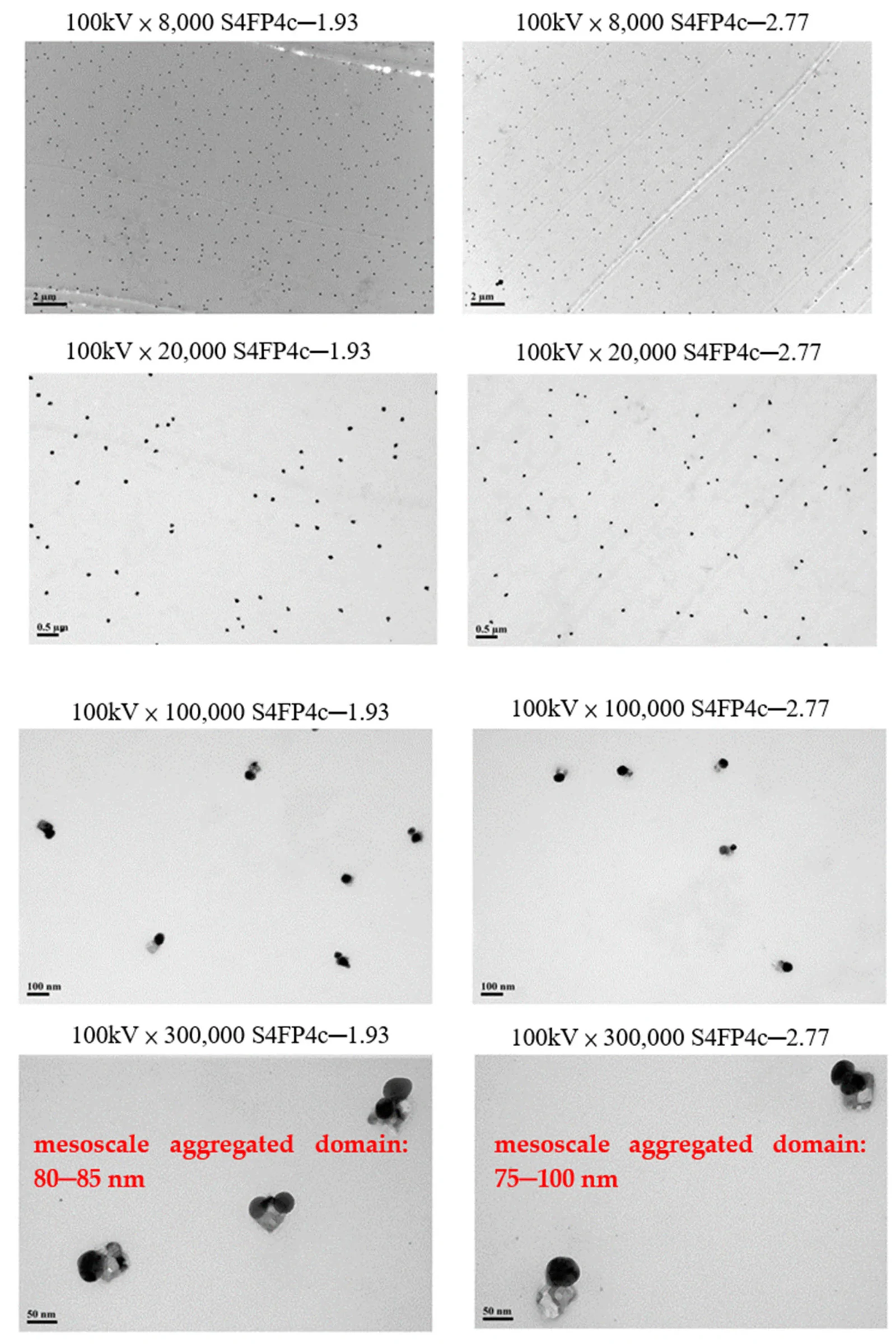
TEM images of S4FP4c series sulfonated polymers.

Introducing fluorine substituents into the polymer structure can significantly increase the contrast between the hydrophilic and hydrophobic segments within the polymer chains, improving the microphase separation morphology of the sulfonated membranes. Additionally, because factors such as reaction time and the concentration of the sulfonating agent during the sulfonation process may cause non-uniform grafting of sulfonic acid groups, the resulting ionic channels formed by the aggregation of sulfonic acid groups might display inconsistent distribution throughout the membrane. Consequently, the TEM images of various materials at the same scale, exhibiting variations in ionic channel size and density, are placed together for comparison. As shown in Figure 25, Figure 26 and Figure 27, the dark regions represent mesoscale aggregated domain formed by the aggregation of hydrophilic sulfonic acid groups. In contrast, the bright regions correspond to hydrophobic fluorine substituents and hydrophobic segments without sulfonic acid grafting. Figure 25, Figure 26 and Figure 27 show the dry-state TEM morphologies of the S4FP4 membranes. The dark features observed in these images are hereafter referred to as sulfonate-rich/hydrophilic domains rather than individual ionic clusters. The apparent dimensions of these domains range from approximately 40 to 150 nm. Importantly, these mesoscale dimensions should not be directly compared with the few-nanometer primary ionic clusters commonly reported for Nafion and other proton-exchange membranes. The TEM features may represent aggregates consisting of multiple smaller ionic domains, and their apparent dimensions can additionally be influenced by specimen preparation and drying. Therefore, the present TEM analysis is primarily used to compare the relative distribution, uniformity, and connectivity of hydrophilic domains among the S4FP4 membranes rather than to determine the intrinsic dimensions of hydrated proton-conducting clusters.

With increasing IEC, the ionic channel size of all S4FP4 series membranes increases; however, the mesoscale aggregated domain density varies minimally among all sulfonated membranes. Figure 25 shows that the S4FP4a (IEC 1.74) membrane possesses a larger mesoscale aggregated domain (100–115 nm) than other sulfonated membranes, and its distribution is more uniform, consistent, and dense. This phenomenon directly correlates with its high proton conductivity (264 mS/cm). Although S4FP4a (IEC 2.80) possesses a higher IEC value, its proton conductivity is lower than that of S4FP4a (IEC 1.74). This is attributed to its non-uniform mesoscale aggregated domain distribution, the presence of numerous gray dead-end (inactive) channels, and a large disparity in mesoscale aggregated domain (50–150 nm). The occurrence of this non-uniform channel distribution and inconsistent sizing is speculated to arise because its degree of sulfonation is close to saturation (82.35%).

Furthermore, Figure 26 shows that the S4FP4b (IEC 1.90) membrane also exhibits non-uniform distribution of mesoscale aggregated domain, with extreme variations in size and distribution density (40–150 nm). Although S4FP4b shows a uniform mesoscale aggregated domain distribution and consistent size, its average mesoscale aggregated domain is the smallest among all membranes evaluated here (60–70 nm). As seen in Figure 27, the two S4FP4c series membranes with different degrees of sulfonation exhibit similar microphase separation morphologies, with very close mesoscale aggregated domain distribution locations and densities; however, a slight difference in mesoscale aggregated domain size is observed. The mesoscale aggregated domain size of S4FP4c (IEC 1.93) is more consistent (80–85 nm), whereas that of S4FP4c (IEC 2.77) shows minor variations (75–100 nm).

The TEM results demonstrate that proton transport is more strongly associated with ionic-domain uniformity and connectivity than with IEC alone. S4FP4a (IEC 1.74) exhibits a relatively narrow ionic-domain distribution of 100–115 nm and achieves σ = 262 mS cm^−1^ and Pmax = 1.07 W cm^−2^. In contrast, S4FP4a (IEC 2.80) exhibits a broader distribution of 50–150 nm with apparent dead-end domains and shows lower values of σ = 214 mS cm^−1^ and Pmax = 0.84 W cm^−2^. Similarly, the relatively uniform 80–85 nm domains of S4FP4c (IEC 1.93), together with low swelling (10.6%) and moderate water uptake (27.8%), enable a Pmax of 0.93 W cm^−2^ despite its lower σ of 124 mS cm^−1^. These comparisons demonstrate that a uniform and effectively hydrated ionic network, while avoiding excessive swelling, is critical for translating ex situ proton conductivity into high in situ fuel-cell performance.

### 4.3. Fuel Cell Performance Testing

**Figure 28:**
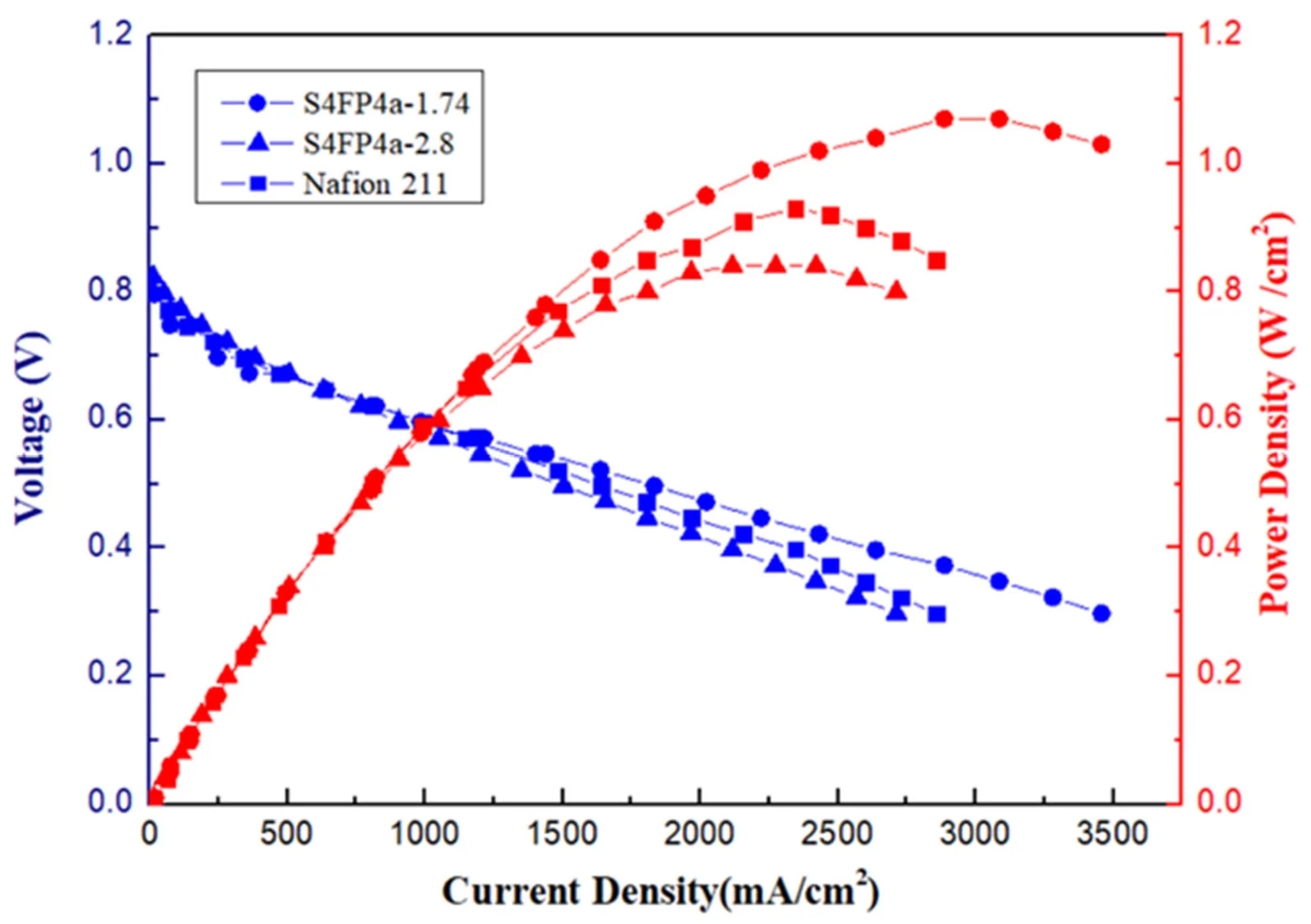
H_2_/O_2_ fuel cell polarization curves of S4FP4a series and Nafion 211 at 80 °C and 100% RH.

**Figure 29:**
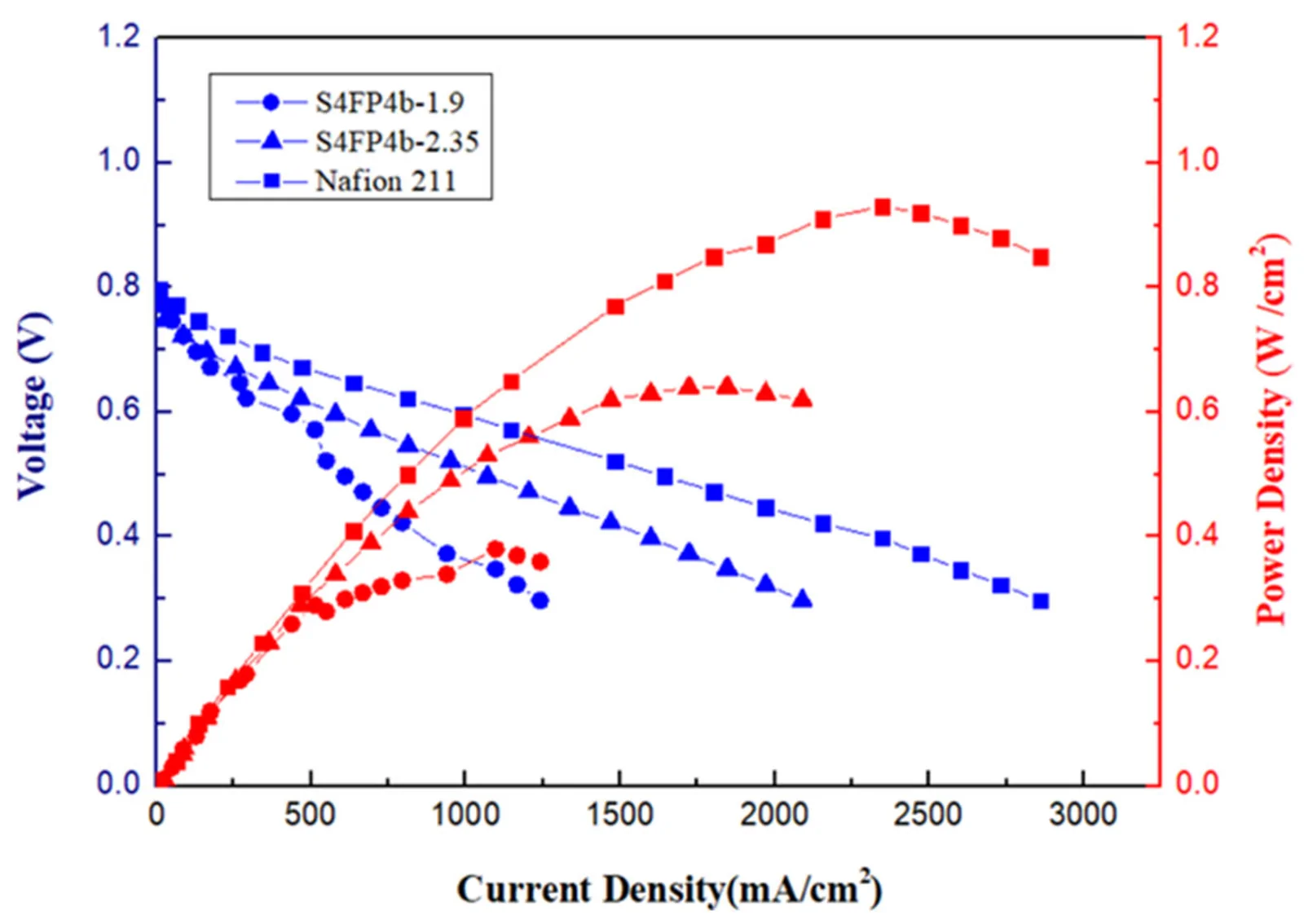
H_2_/O_2_ fuel cell polarization curves of S4FP4b series and Nafion 211 at 80 °C and 100% RH.

**Figure 30:**
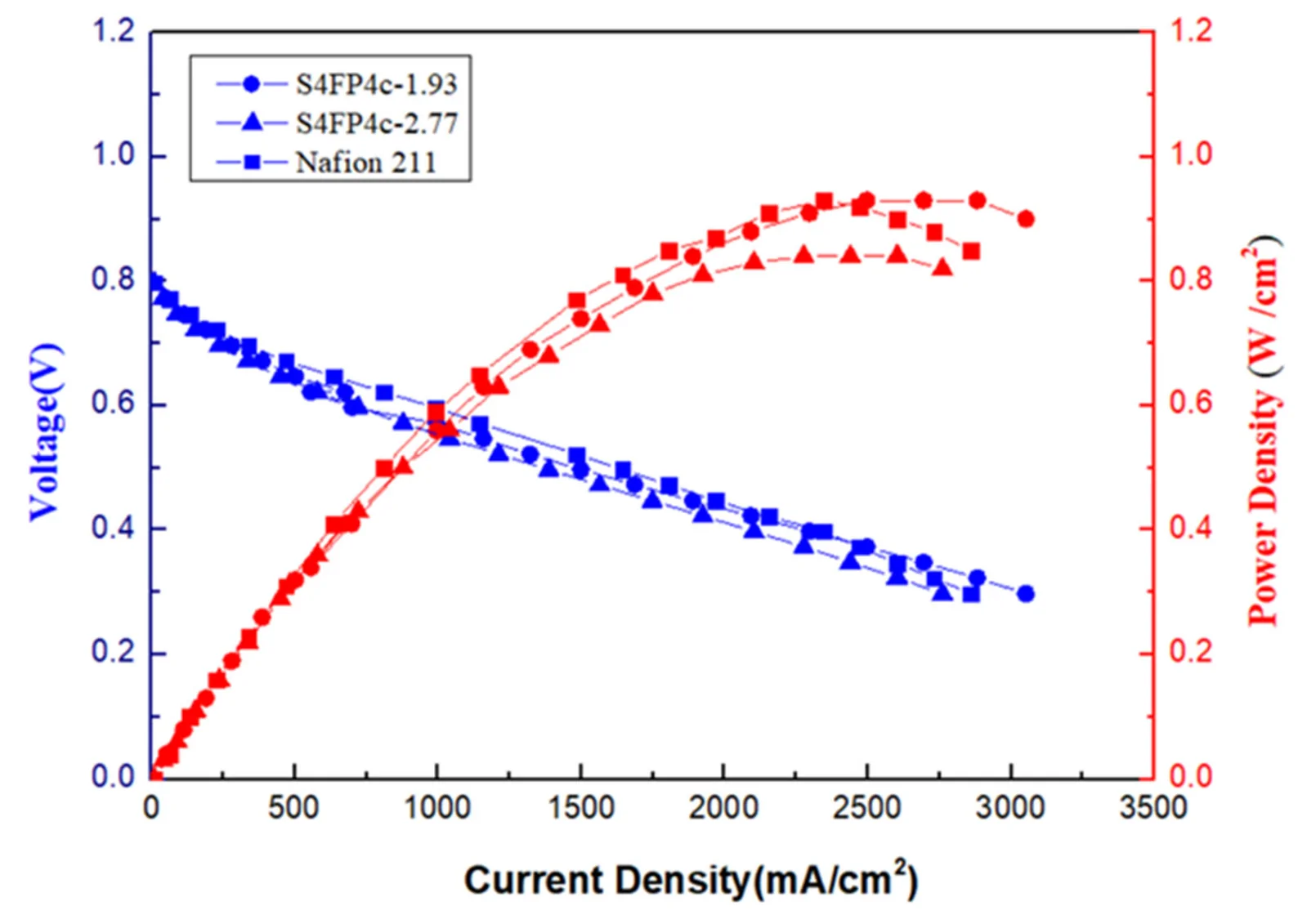
H_2_/O_2_ fuel cell polarization curves of S4FP4c series and Nafion 211 at 80 °C and 100% RH.

Figure 28, Figure 29 and Figure 30 display the fuel cell polarization curves of the S4FP4 series and the commercial material Nafion 211 at 80 °C and 100% RH using hydrogen and oxygen as the fuel. As shown in Figure 28, the open-circuit voltage of S4FP4a (IEC 1.74) is 0.822 V, and its maximum fuel cell power density reaches up to 1.07 W/cm^2^, representing the highest cell performance among the S4FP4 series membranes and outperforming Nafion 211 (0.93 W/cm^2^). This superior cell performance is presumably attributed to the high proton conductivity (262 mS/cm), excellent dimensional stability (<17%), and appropriate water uptake (65%) of S4FP4a (IEC 1.74). In contrast, S4FP4a (IEC 2.8) exhibits a lower cell performance of only 0.84 W/cm^2^, which is related to its non-uniform ionic channel distribution.

As illustrated in Figure 29, although S4FP4b (IEC 2.35) possesses a decent proton conductivity (202 mS/cm), its maximum fuel cell performance is only 0.64 W/cm^2^. This poor performance arises from its relatively inferior dimensional stability (26%) and excessively high water uptake (116%), which lead to excessive swelling and deformation of the membrane during fuel cell operation. Furthermore, Figure 30 demonstrates that the open-circuit voltages of both S4FP4c (IEC 1.93) and S4FP4c (IEC 2.77) are 0.798 V, while their maximum power densities reach 0.93 W/cm^2^ and 0.84 W/cm^2^, respectively. Although S4FP4c (IEC 1.93) has a lower IEC value and poorer proton conductivity (124 mS/cm), its improved cell performance is enabled by its superior microphase separation morphology, dimensional stability (10.6%), and moderate water uptake (27.8%) compared to S4FP4c (IEC 2.77).

Notably, in the cell performance analysis, S4FP4c (IEC 1.93) displays a polarization curve similar to that of Nafion 211. This similarity stems from their comparable membrane characteristics and proton conductivities (124 mS/cm versus 123.8 mS/cm). Moreover, data analysis of their fundamental membrane properties across different temperatures shows negligible differences, indicating low temperature dependence for both, which ultimately leads to their close fuel cell performance.

In summary, the cell performance data demonstrate that a high proton conductivity does not inherently guarantee outstanding fuel cell efficiency. For a sulfonated membrane to achieve exceptional fuel cell performance, it requires not only good electrical properties but also favorable microphase separation morphology, dimensional stability, thermal stability, and appropriate water uptake. In other words, the chemical, physical, thermal, mechanical, electrical, and microphase morphological properties of the membrane must be well-matched and balanced to ensure excellent efficiency when applied in fuel cells [36]. The original manuscript, as presented in Figure 28, Figure 29 and Figure 30, presents representative single-cell polarization measurements. However, the original data lacks sufficient independent MEA replication experiments for statistically significant standard deviation analysis. Therefore, we do not wish to include extrapolated error bars without experimental evidence. To avoid overstating experimental reproducibility, we have designated repeatability testing and statistical uncertainty analysis for different MEAs as future research areas.

The literature values (Table 10) are consistent with the reported data for the partially fluorinated s-P12F97B system, BM-1 blend membrane, and SFC2-2.53 membrane.

## 5. Conclusions

Although IEC reflects the concentration of ionizable sulfonic acid sites, it does not directly represent the connectivity and effective hydration of proton-conducting pathways. S4FP4a (IEC 1.74) combines a relatively narrow ionic-domain size distribution (100–115 nm), a highly uniform domain distribution, appropriate water uptake (65% at 90 °C), and limited dimensional swelling (17%). These characteristics facilitate efficient and continuous proton transport while maintaining membrane integrity. In contrast, the higher-IEC S4FP4a (IEC 2.80) exhibits a broad ionic-domain distribution (50–150 nm) with apparent dead-end domains, resulting in lower effective proton conductivity and fuel-cell performance. Therefore, fuel-cell performance is governed by the combined effects of proton conductivity, effective channel connectivity, hydration state, water management, and dimensional integrity rather than by IEC alone.

The ion exchange capacity (IEC) values of the S4FP4 series range between 1.74 and 2.8 mmol/g, with 5% weight loss temperatures ($T_d5%_) spanning from 238 to 259 °C. With respect to water uptake and dimensional stability, all sulfonated polymers (S4FP4 series) maintain an intact membrane morphology under a high-temperature and high-humidity environment at 90 °C. S4FP4b (IEC 2.35) preserves excellent dimensional stability (<26%) even under a high water uptake of 116.3%. Because a single fluorine substituent exhibits weaker hydrophobicity than a trifluoromethyl (–CF_3_) group, its incorporation into the polymer backbone not only effectively enhances dimensional stability while maintaining appropriate water uptake, but also avoids severely restricting the degree of sulfonation, thereby retaining desirable IEC values.

The previously developed SP4 membranes exhibited high proton conductivity, but their excessive water uptake caused severe swelling and, at relatively high IEC values, even dissolution under humid conditions. In contrast, the earlier FP systems containing a high proportion of strongly hydrophobic –CF_3_ groups exhibited improved dimensional stability, but excessive fluorination could restrict sulfonation, water uptake, and proton transport. The present S4FP4 design provides an intermediate and better-balanced strategy. The additional mono-fluorine substituents improve hydrophobic–hydrophilic contrast and dimensional stability without suppressing sulfonation and hydration as strongly as excessive –CF_3_ substitution. As a result, S4FP4a-1.74 simultaneously achieved a proton conductivity of 262 mS cm^−1^ at 80 °C and 95% RH, a dimensional swelling of 17% at 90 °C, and a maximum power density of 1.07 W cm^−2^. These combined properties represent the principal scientific advancement of the present membrane design.

Experimental data indicate that incorporating fluorine substituents into the polymer structure effectively increases the proton conductivity of the membranes. This enhancement is attributed to the fact that the introduction of fluorine effectively directs the grafting positions of the sulfonic acid groups, yielding more distinct hydrophilic and hydrophobic segments. This well-defined structure facilitates a superior microphase-separated morphology, significantly boosting the proton conductivity. Furthermore, although S4FP4a (IEC 1.74) possesses a lower IEC value than S4FP4a (IEC 2.80), it exhibits a higher proton conductivity (262 mS/cm) and a superior peak power density (1.07 W/cm^2^). Despite having fewer sulfonic acid groups, S4FP4a (IEC 1.74) benefits from highly consistent mesoscale aggregated domain, enhanced mesoscale aggregated domain distribution uniformity, and optimal mesoscale aggregated domain, which ultimately culminate in an optimized microphase separation morphology.

In conclusion, introducing fluorine substituents into the polymer matrix successfully amplifies the contrast between the hydrophilic and hydrophobic domains within the polymer chains, refining the microphase separation morphology of the membranes. Consequently, all developed membranes achieve simultaneous enhancements in both proton conductivity and overall membrane stability, translating into outstanding cell performance during single-cell fuel cell testing.

Although the S4FP4 membranes exhibited excellent ex situ oxidative and hydrolytic stability, long-term in situ durability under continuous PEMFC operation was not evaluated in the present study. Future work will therefore focus on extended constant-current or constant-voltage operation, voltage-decay measurements, relative-humidity cycling, and start–stop cycling to evaluate membrane degradation and membrane–electrode assembly durability under practical operating conditions.

## Figures and Tables

**Table 1:** Sulfonating reagents at different concentrations.

Reaction Scale	Chlorosulfonic Acid	Lauric Acid
3.25 mL	3.25 mL (5.671 g)	11.7 g
2.5 mL	2.5 mL (4.3625 g)	9.75 g

**Table 2:** Degree of sulfonation of the polymers.

Polymer	Chlorosulfonic Acid (mL)	IEC (*tit.*) (mmol/g) ^a^	IEC (*theo.*) (mmol/g) ^b^	Degree of Sulfonation (%) ^c^
S4FP4a-1.74	2.5	1.74	3.4	51.17
S4FP4a-2.8	3.25	2.8	3.4	82.35
S4FP4b-1.9	2.5	1.9	3.8	50
S4FP4b-2.35	3.25	2.35	3.8	61.84
S4FP4c-1.93	2.5	1.93	3.8	50.78
S4FP4c-2.77	3.25	2.77	3.8	72

^a^. Measured IEC value. ^b^. Theoretical IEC value. ^c^. Ratio of the measured IEC to the theoretical IEC. IEC (theo.): Theoretical value and IEC (tit.): Measured value from titration.

**Table 3:** GPC data analysis of the H_2_FA and HFA series.

Polymer	Mw (g/mol)	Mn (g/mol)	PDI (Mw/Mn)	Film-Forming Ability
**4FP4a**	182,057	134,034	1.358	Good
**4FP4b**	168,583	144,789	1.164	Good
**4FP4c**	42,090	40,903	1.029	Good

**Table 4:** T_d5%_ values of the polymers.

	4FP4a	4FP4b	4FP4c
**T_d5%_ (°C)**	552	553	534

**Table 5:** Thermal decomposition temperatures of the sulfonated polymers.

	S4FP4a (IEC 1.74)	S4FP4a (IEC 2.80)
T_d5%_ (°C)	259	256
	S4FP4b (IEC 1.9)	S4FP4b (IEC 2.35)
T_d5%_ (°C)	251	254
	S4FP4c (IEC 1.93)	S4FP4c (IEC 2.77)
T_d5%_ (°C)	238	247

**Table 6:** Basic properties of the sulfonated polymer membranes.

Sulfonated Polymer	IEC (mmol/g)	Water Uptake (%)		Dimensional Swelling (%)		Hydration Number (λ)	
		30 °C	90 °C	30 °C	90 °C	30 °C	90 °C
**S4FP4a (IEC 1.74)**	1.74	35	65	11	17	11.1	20.7
**S4FP4a (IEC 2.80)**	2.80	33.4	49	9.75	11.2	6.6	9.7
**S4FP4b (IEC 1.90)**	1.90	16	24.7	2	2.58	4.67	7.2
**S4FP4b (IEC 2.35)**	2.35	57.6	116.3	21	26.3	13.6	27.5
**S4FP4c (IEC 1.93)**	1.93	23.3	27.8	7.05	10.6	6.7	8
**S4FP4c (IEC 2.77)**	2.77	37.1	78.5	17	23	7.4	15.7
**Nafion 211**	0.91	14	36	14	31.4	9	22

**Table 7:** Short-term oxidative resistance of the sulfonated polymer membranes after 1 h exposure to Fenton’s reagent at 80 °C.

Polymer	Oxidative Stability (%)
S4FP4a (IEC 1.74)	97.7
S4FP4a (IEC 2.80)	100.0
S4FP4b (IEC 1.90)	100.0
S4FP4b (IEC 2.35)	97.6
S4FP4c (IEC 1.93)	98.3
S4FP4c (IEC 2.77)	96.4
Nafion 211	98.0

**Table 8:** Hydrolytic stability of the sulfonated polymers.

Polymer	Hydrolytic Stability(%) ^a^
S4FP4a (IEC 1.74)	**99**
S4FP4a (IEC 2.80)	100
S4FP4b (IEC 1.90)	99
S4FP4b (IEC 2.35)	100
S4FP4c (IEC 1.93)	100
S4FP4c (IEC 2.77)	99
Nafion 211	99

^a^. In 100 °C DI water for 24 h.

**Table 9:** Proton conductivity of sulfonated polymers at a fixed temperature of 80 °C under various relative humidities.

Sulfonated Polymer	Proton Conductivity at 80 °C (mS/cm)			
	40% RH	60% RH	80% RH	95% RH
**S4FP4a (IEC 1.74)**	22	34	117	262
**S4FP4a (IEC 2.80)**	17	28	109	214
**S4FP4b (IEC 1.90)**	14	16	20	33
**S4FP4b (IEC 2.35)**	15	33	113	202
**S4FP4c (IEC 1.93)**	11	24	59	124
**S4FP4c (IEC 2.77)**	12	36	103	209
**Nafion 211**	14.6	38.8	64.5	123.8

**Table 10:** Comparison of the present S4FP4a membrane with representative high-performance hydrocarbon proton exchange membranes.

Membrane	IEC (mmol g^−1^)	Water Uptake	Dimensional Swelling	Proton Conductivity	Peak Power Density
S4FP4a (IEC 1.74)	1.74	65% (90 °C)	17% (90 °C)	262 mS cm^−1^ (80 °C, 95% RH)	1.07 W cm^−2^ (80 °C, 100% RH)
s-P12F97B-2.92	2.92	78.6% (80 °C)	ΔA = 43.1%; ΔT = 42.5% (80 °C)	187.3 mS cm^−1^ (80 °C, 95% RH)	1.32 W cm^−2^
BM-1	2.89	91.2% (80 °C)	ΔA = 34.5% (80 °C)	191.2 mS cm^−1^ (80 °C, 95% RH)	1.30 W cm^−2^
SFC2-2.53	2.53	47.3% (80 °C)	ΔL = 27.8%; ΔT = 56.5% (80 °C)	240 mS cm^−1^ (80 °C, 95% RH)	1.41 W cm^−2^
Nafion 211, this work	0.91	36% (90 °C)	31.4% (90 °C)	123.8 mS cm^−1^ (80 °C, 95% RH)	0.93 W cm^−2^

Note: The definitions and temperatures used for dimensional swelling differ among the literature reports; therefore, the values should be interpreted as comparative benchmarks rather than strictly equivalent test results.

## Data Availability

The raw data supporting the conclusions of this article will be made available by the authors on request.
